# Multiple *Campylobacter jejuni* proteins affecting the peptidoglycan structure and the degree of helical cell curvature

**DOI:** 10.3389/fmicb.2023.1162806

**Published:** 2023-04-18

**Authors:** Emilisa Frirdich, Jenny Vermeulen, Jacob Biboy, Waldemar Vollmer, Erin C. Gaynor

**Affiliations:** ^1^Department of Microbiology and Immunology, University of British Columbia, Vancouver, BC, Canada; ^2^The Centre for Bacterial Cell Biology, Institute for Cell and Molecular Biosciences, Newcastle University, Newcastle upon Tyne, United Kingdom

**Keywords:** *Campylobacter jejuni*, bacterial cell shape, helical morphology, morphological quantitation, peptidoglycan, bactofilin, M23 peptidase domain, peptidoglycan hydrolase

## Abstract

*Campylobacter jejuni* is a Gram-negative helical bacterium. Its helical morphology, maintained by the peptidoglycan (PG) layer, plays a key role in its transmission in the environment, colonization, and pathogenic properties. The previously characterized PG hydrolases Pgp1 and Pgp2 are important for generating *C. jejuni* helical morphology, with deletion mutants being rod-shaped and showing alterations in their PG muropeptide profiles in comparison to the wild type. Homology searches and bioinformatics were used to identify additional gene products involved in *C. jejuni* morphogenesis: the putative bactofilin 1104 and the M23 peptidase domain-containing proteins 0166, 1105, and 1228. Deletions in the corresponding genes resulted in varying curved rod morphologies with changes in their PG muropeptide profiles. All changes in the mutants complemented except *1104*. Overexpression of *1104* and *1105* also resulted in changes in the morphology and in the muropeptide profiles, suggesting that the dose of these two gene products influences these characteristics. The related helical ε-Proteobacterium *Helicobacter pylori* has characterized homologs of *C. jejuni* 1104, 1105, and 1228 proteins, yet deletion of the homologous genes in *H. pylori* had differing effects on *H. pylori* PG muropeptide profiles and/or morphology compared to the *C. jejuni* deletion mutants. It is therefore apparent that even related organisms with similar morphologies and homologous proteins can have diverse PG biosynthetic pathways, highlighting the importance of studying PG biosynthesis in related organisms.

## 1. Introduction

The Gram-negative bacterium *Campylobacter jejuni* is an important zoonotic pathogen that can cause severe disease in humans while asymptomatically colonizing the intestinal tracts of birds and other animals (Young et al., 2007; Bolton, 2015; Burnham and Hendrixson, 2018; Tegtmeyer et al., 2021). *Campylobacter jejuni* is a major cause of bacterial gastroenteritis worldwide, often outnumbering the more widely known and studied enteric pathogens *Escherichia coli*, *Salmonella*, and *Shigella* in the developed world (Sherman et al., 2010; Silva et al., 2011; Kaakoush et al., 2015; Sheppard and Maiden, 2015; Hansson et al., 2018). Evidence also suggests a global rise in *C. jejuni* infections over the last decade (Kaakoush et al., 2015).

The name *Campylobacter* means “curved rod,” derived from the Greek campylos (curved) and baktron (rod) describing the predominant morphology of *C. jejuni* in its most pathogenic form. As *C. jejuni* ages, and under stress and adverse growth conditions, it can adopt different morphologies such as that of a filamented helical rod (Justice et al., 2008; Cameron et al., 2012; Frirdich and Gaynor, 2013) and/or a coccoid form (Svensson et al., 2008; Jackson et al., 2009; Ikeda and Karlyshev, 2012; Frirdich et al., 2019). Rod-shaped variants have also been detected in *C. jejuni* populations by passage through the chicken gut or chick embryos (Field et al., 1993; Hänel et al., 2009), by laboratory passage (Esson et al., 2016; Frirdich et al., 2017), and by selection through laboratory exposure to the compound calcofluor white (CFW; Frirdich et al., 2017). Changes to the *C. jejuni* helical shape affect its biological properties and pathogenic potential (Cameron et al., 2012; Frirdich et al., 2012, 2014, 2019; Ha et al., 2016; Stahl et al., 2016), altering its ability to be transmitted, colonize, and interact with its host, all of which ultimately impact disease outcome. Therefore, studying *C. jejuni* morphology determination is a key aspect of understanding *C. jejuni* pathogenesis and survival.

Bacterial cell shape is maintained by the peptidoglycan (PG) sacculus (Vollmer et al., 2008a). PG is a macromolecular structure essential in most bacteria, including *C. jejuni*. It surrounds the cytoplasmic membrane to form a rigid mesh-like layer that protects the cell from lysis due to turgor, as well as maintaining morphology. The basic composition of PG is generally conserved among bacteria, consisting of glycan chains of β-1-4 linked N-acetylglucosamine (GlcNAc) and N-acetylmuramic acid (MurNAc) residues cross-linked by short peptides. Glycan strands terminate with 1,6-anhydro MurNAc (anhMurNAc) residues. In Gram-negative bacteria, the peptides primarily consist of L-Ala_1_-D-iso-glutamate_2_-mesodiaminopimelic acid (mDAP)_3_-D-Ala_4_-D-Ala_5_ residues and are attached to the MurNAc residue. Most cross-links between peptides of adjacent strands occur between the mDAP_3_ of one stem peptide and D-Ala_4_ of another. No other cross-links were detected in *C. jejuni* (Frirdich et al., 2012). PG structure shows a high diversity between bacterial species but is relatively well conserved within a species (Vollmer et al., 2008a; Pazos and Peters, 2019). However, variations within a species do occur depending on the strain, growth conditions, and growth phase (Vollmer, 2008; Pazos and Peters, 2019). In addition, bacteria can modulate their PG, and these structural modifications can serve as a protection strategy against environmental threats such as those posed by predatory PG hydrolytic proteins and antimicrobial compounds (Vollmer et al., 2008a; Yadav et al., 2018; Pazos and Peters, 2019). These variations can include differences in the length of the glycan strand, length and composition of the stem peptide, degree and type of cross-linking, and the presence or absence of modifications (Vollmer et al., 2008a; Yadav et al., 2018; Pazos and Peters, 2019).

Growth of the PG layer involves the highly coordinated activity of synthases and hydrolases regulated by factors such as cytoskeletal elements (Typas et al., 2012; Egan et al., 2020). PG subunits are incorporated into the growing glycan chain by transglycosylases; then, inter-strand peptide cross-linking is formed by transpeptidases. *C. jejuni* has a homolog of the PBP1a (CJJ811876_0536) synthetase, as well as homologs of the transpeptidases PBP2 (CJJ811876_0680; involved in cell elongation) and PBP3 (CJJ811876_0550; involved in cell division). PG hydrolases play an important role in PG biosynthesis by cleaving PG bonds and allowing insertion of new PG subunits during growth and elongation, ensuring cell separation during division, and remodeling the PG to generate shape (Vollmer et al., 2008b; Uehara and Bernhardt, 2011; Wyckoff et al., 2012; Frirdich and Gaynor, 2013; Cava and de Pedro, 2014; Do et al., 2020). This process is key in bacterial differentiation, enabling bacteria to adapt to different environmental conditions (Vollmer et al., 2008b; Wyckoff et al., 2012; Frirdich and Gaynor, 2013; Cava and de Pedro, 2014; Do et al., 2020). PG hydrolases also have numerous other functions such as spore maturation, roles in PG recycling, and as phage and predator lysins (Vollmer et al., 2008b; Wyckoff et al., 2012; Frirdich and Gaynor, 2013; Cava and de Pedro, 2014; Do et al., 2020). Differences in the complement of PG hydrolases between bacterial species contribute to variations in the muropeptide profile between organisms, can influence the morphology, and can impact overall bacterial fitness and pathogenesis.

Peptidoglycan hydrolases are classified based on their cleavage specificity (Vollmer et al., 2008b; Vermassen et al., 2019). Glycosidases cleave the PG backbone. Amidases cleave the peptide from the glycan backbone, while peptidases cleave within the peptides. One amidase, AmiA, has been identified in *C. jejuni* (Frirdich et al., 2019). Peptidases can be further classified into carboxypeptidases that cleave the C-terminal peptide and endopeptidases that cleave within the peptides. Three PG peptidases have been characterized in *C. jejuni*: Pgp1, Pgp2, and Pgp3, which are all involved in helical morphology generation (Frirdich et al., 2012, 2014; Frirdich and Gaynor, 2013; Min et al., 2020). Pgp1 is a DL carboxypeptidase that cleaves PG tripeptides to dipeptides. We identified Pgp1 as a hypo-reactive mutant in a transposon mutant screen using calcofluor white (CFW), a fluorescent dye binding β-1-3 and β-1-4 linked sugars such as those found in PG (Frirdich et al., 2012). Altered CFW reactivity correlates with changes in *C. jeuni* pathogenic properties (McLennan et al., 2008). We identified Pgp2 bioinformatically by STRING analysis which suggested Pgp2 as a potential interaction partner for Pgp1. Further analyses showed that Pgp2 is an LD-carboxypeptidase cleaving PG tetrapeptides to tripeptides, providing the substrate for Pgp1 (Frirdich et al., 2014). No additional PG hydrolases were identified using these methods. Deletion mutants in *pgp1* and *pgp2* were rod-shaped and had an altered PG muropeptide profile. Pgp3, characterized by Min et al. (2020), was shown to have DD-carboxypeptidase (cleaving penta- to tetra-peptides) and DD-endopeptidase activity (cleaving tetra-tri peptide cross-links) using synthetic PG substrates, with the DD-endopeptidase activity also having been demonstrated on *E. coli* sacculi (Min et al., 2020). A ∆*pgp3* mutant has a curved rod appearance, but changes in the mutant PG profile have yet to be determined.

In this study, bioinformatic approaches were used to identify additional *C. jejuni* PG hydrolases CJJ81176_0166 (henceforth referred to by the gene number, 0166), CJJ81176_1105 (1105), CJJ81176_1228 (1228), and the bactofilin CJJ81176_1104 (1104). The roles of these proteins in the maintenance of *C. jejuni* helical morphology and in PG biosynthesis were analyzed by examining the morphology and muropeptide profiles of mutant, complement, and overexpressing strains. Deletion of *0166*, *1104*, *1105*, and *1228* produced strains with morphologies varying in curvature in comparison to the wild type. Unlike mutants in *C. jejuni* PG hydrolases *pgp1* and *pgp2*, analysis of the mutant muropeptide profile of ∆*0166*, ∆*1105*, and ∆*1228* displayed complex changes. In addition, overexpression with 1104 resulted in a more pronounced loss of curvature, while overexpression with 1105 resulted in a gain in curvature, both accompanied by muropeptide changes. The broad effects that the loss and/or overexpression of *0166*, *1104*, *1105*, and *1228* have on the muropeptide composition, highlighted by analyses of double and triple mutants, support the idea that the presence and levels of these gene products have effects on each other and/or other PG biosynthetic proteins.

Like *C. jejuni*, *Helicobacter pylori* is a member of the ε-Proteobacteria and has a helical morphology. *H. pylori* has characterized homologs of 1104 (CcmA), 1105 (Csd1), and 1228 (Csd3/HdpA; henceforth referred to as Csd3 unless the specific *hdpA* mutant or HdpA protein is being referred to; Bonis et al., 2010; Sycuro et al., 2010). However, deletions in these homologs had differing morphological and/or muropeptide changes to the *C. jejuni* proteins (Sycuro et al., 2010). This indicates that the morphogenesis program of *C. jejuni* and *H. pylori* has unique differences, despite being related organisms with similar helical morphology and several homologous enzymes. Therefore, it is important to study the PG biosynthetic program in related bacterial pathogens and the biological role of homologous PG enzymes.

## 2. Materials and methods

### 2.1. Bacterial strains and growth conditions

The bacterial strains and plasmids used in this study and their construction are described in the Supplementary Information. All strains were verified by PCR and sequencing. Unless otherwise indicated, *C. jejuni* strains were grown at 38°C in Mueller–Hinton (MH; Oxoid) broth or on 1.7% (w/v) agar plates supplemented with vancomycin (10 μg/ml) and trimethoprim (5 μg/ml), denoted MH-TV, under microaerobic/capnophilic conditions (6% O_2_, 12% CO_2_) in a Sanyo tri-gas incubator for plates or using the Oxoid CampyGen system for broth cultures. Growth media were supplemented with chloramphenicol (Cm; 20 μg/ml), kanamycin (Km; 50 μg/ml), or apramycin (Apr; 60 μg/ml) where appropriate. *E. coli* strains used for plasmid construction were grown at 38°C in Luria–Bertani (LB; Sigma) broth or 1.5% agar (w/v) agar plates and supplemented with ampicillin (Ap, 100 μg/ml), chloramphenicol (15 μg/ml), kanamycin (25 μg/ml), or apramycin (60 μg/ml) as necessary.

For growth analyses, *C. jejuni* strains were streaked from 16- to 18-h plate cultures and grown again on plates for 7–8 h. Bacteria were harvested in MH-TV broth and inoculated at an OD_600_ of 0.0005 into MH-TV broth and grown shaking for 18 h. Strains were subcultured to an OD_600_ of 0.005, and samples were taken at various timepoints for the colony-forming unit (CFU) and microscopic analysis. For morphological analysis, overnight cultures were subcultured to an OD_600_ of 0.05 and incubated for 4 h to generate mid-exponential phase cultures.

### 2.2. Microscopy and morphological analysis

Visualization under DIC microscopy and TEM was carried out as described (Frirdich et al., 2019). In brief, for visualization under DIC microscopy, 1 μl of a mid-exponential phase broth culture was immobilized on a thin 1% agar (*w*/*v* in H20) slab and overlayed with a cover slip. Images were captured with a Nikon Eclipse TE2000-U microscope equipped with 100 × objective and a Hamamatsu Orca camera system. DIC images were taken from multiple fields of view (totaling approximately 300 cells/strain). Images were converted to an 8-bit gray scale and processed using FIJI by applying a median filter of radius 1. Binary images were thresholded (Pincus and Theriot, 2007) using a pixel block size of 102 and subtracting −4. Artifacts and cells that were cut off on the sides of the image were manually removed. Morphological analysis was carried out using the MicrobeJ plug-in for Fiji (Ducret et al., 2016) and the CellTool shape analysis program (Pincus and Theriot, 2007; Sycuro et al., 2010; Ha et al., 2016). Both programs enable automated high throughput processing of binary bacterial images to detect bacterial cell contours at sub-pixel resolution and analyze bacterial cell shape parameters. Contours were generated in MicrobeJ using the medial axis method. These were analyzed, and a result table of the values of the shape descriptors was generated. The mean values and standard deviations were calculated, and significant differences were determined using multiple comparisons with Dunn’s correction after a Kruskal–Wallis test using GraphPad Prism 8. CellTool was used to compare differences in population morphology using the principal component analysis (PCA), as well as differences in the distributions of axis length, normalized axis curvature, and side curvature. CellTool analysis was carried out as previously described (Pincus and Theriot, 2007). For PCA, an average *C. jejuni* wild-type cell shape was generated by aligning the contours of the wild-type population. PCA was performed to generate a shape model based on principal components called shape modes. The shape modes combined describe at least 94.9% of the variation in the wild-type population. Contours of other strains were then aligned using the wild-type PCA shape model as a reference. The Kolmogorov–Smirnov statistics were calculated to determine whether changes between the different strains and wild type were significantly different with significance denoted by *p*-values of < 0.05.

### 2.3. Peptidoglycan isolation and muropeptide analysis

*Campylobacter jejuni* strains were passaged once from frozen stocks and then passaged to 20–25 MH plates and grown for 18–20 h. Cells were collected into cold MH broth by scraping to a total amount equivalent to an OD of 200–600, harvested by centrifugation at 8000 ×*g* for 15 min, and then resuspended in 6 ml ice-cold H_2_O. Cells were lysed by dropwise addition to an equivalent amount of 8% SDS to that of the cell suspension boiling under reflux. PG was purified from the cell lysate, digested with the muramidase cellosyl (kindly provided by Hoechst, Frankfurt, Germany), and the resulting muropeptides were reduced with sodium borohydride and separated by HPLC as described (Glauner, 1988). Muropeptide structures were assigned based on a comparison with retention times of known muropeptides from *C. jejuni* (Frirdich et al., 2012).

## 3. Results and discussion

### 3.1. Identification and bioinformatics analysis of proteins with a role in *Campylobacter jejuni* morphology

**Figure 1:**
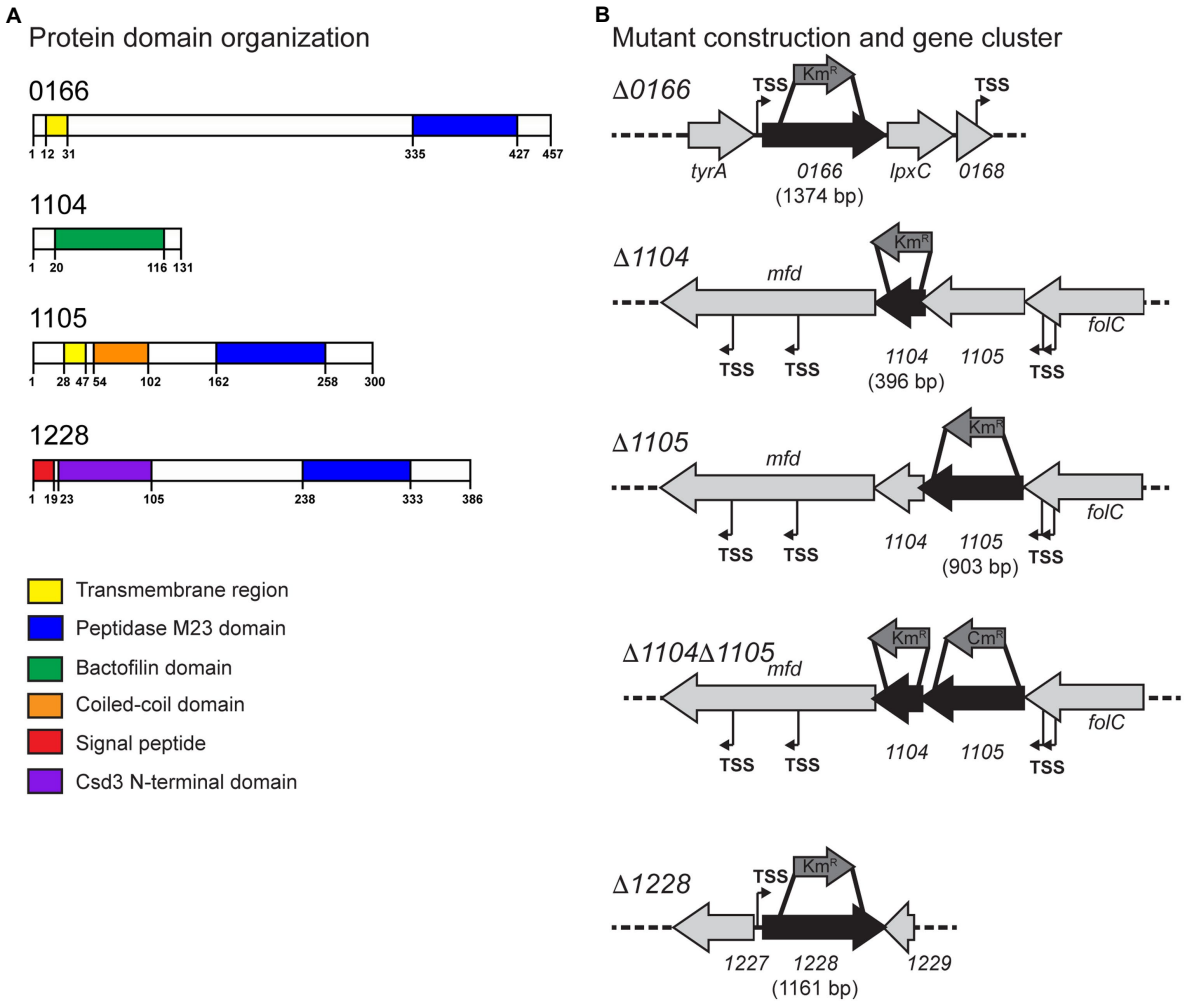
Protein domains of *Campylobacter jejuni* 0166, 1104, 1105, and 1228 and the corresponding gene loci. **(A)** The protein domains in the *0166*, *1104*, *1105*, and *1228* gene products predicted by jackhmmer. **(B)** The gene loci of *0166*, *1104*, *1105*, *1104*–*1105*, and *1228* and the approximate location of the internal fragments deleted and replaced with a non-polar Km^R^ cassette or a Cm^R^ cassette for mutant construction. Putative promoters (transcription start sites, TSS) predicted by RNA-seq are indicated (Dugar et al., 2013).

Gene products with a potential role in *C. jejuni* cell shape determination were identified by two bioinformatic approaches: (1) homology searches with characterized *H. pylori* PG hydrolases and cytoskeletal elements (Sycuro et al., 2010), and (2) searching the genome of *C. jejuni* strain 81-176 for proteins with peptidase domains. Those identified by approach (2) were further narrowed down to peptidases within the MEROPS M23B metallopeptidase family, some of which have a known role in PG biosynthesis (Vermassen et al., 2019). Approach (1) identified the genes *cjj81176_1104* (*1104*), *cjj81176_1105* (*1105*), and *cjj81176_1228* (*1228*), and approach (2) identified *cjj81176_0166* (*0166*) in addition to *1105* and *1228* (Figure 1).

#### 3.1.1. M23 peptidase domain-containing protein CJJ81176_0166

Conserved domain analysis of the *0166* gene product (457 amino acids) by jackhmmr (Potter et al., 2018) identified a transmembrane region (amino acids 12-31) and an M23 peptidase domain (PF01551; amino acids 335-427) with the conserved metalloprotease HxxxD and HxH motifs (Figure 1A). The N-terminal domain exhibited no similarities to other proteins or conserved domains. 0166 showed 27% identity/46% similarity to the uncharacterized *H. pylori* 26695 protein HP1054. Uncharacterized homologs of 0166 are well conserved in species of the δ- and ε-bacteria and Spirochaetia, all of which primarily consist of bacteria with curved or helical morphologies.

#### 3.1.2. CJJ81176_1104 bactofilin homolog

Thousand hundred and four has 36.4% identity/59.3% similarity to CcmA of *H. pylori* 2695 HP1542.

As with *H. pylori* CcmA (Sycuro et al., 2010; Sichel et al., 2022), 1104 (131 amino acids) has a bactofilin domain (PF04519, amino acids 20-116; Figure 1A; Supplementary Figure S1). Bactofilins are small β-helical proteins that form cytoskeletal filaments (Kühn et al., 2010). They are found in a wide range of bacteria and have diverse functions, including modulating cell shape in *H. pylori* (Sycuro et al., 2010; Taylor et al., 2020; Sichel et al., 2022). *H. pylori* CcmA localizes to the major axis of curvature and helps to recruit the PG synthesis machinery and coordinate the modifications required to generate helical morphology (Taylor et al., 2020; Sichel et al., 2022). The bactofilin domain is flanked by an N-terminal and C-terminal region in *H. pylori*, an organization that is conserved in 1104 (Supplementary Figure S1). The N-terminal region contains a highly conserved short membrane binding motif (Deng et al., 2019; Supplementary Figure S1). This motif was shown to mediate membrane binding in the *Thermus thermophilus* bactofilin but not in *H. pylori* CcmA (Deng et al., 2019; Sichel et al., 2022). The *H. pylori* CcmA N-terminal region is important in generating helical cell shape and regulating interactions with the transmembrane scaffolding protein Csd7 (Sichel et al., 2022). The *C. jejuni* 1104 N-terminal domain will function differently, as *C. jejuni* lacks a Csd7 homolog, and may thus have more of a membrane binding role. The bioinformatic STRING analysis showed a potential interaction between the 1104 and the 1105 putative peptidase (described below) and vice versa based on their co-occurrence. *H. pylori* CcmA and Csd1/2 (1105 homologs) do not interact directly, but CcmA does regulate Csd1 activity indirectly (Yang et al., 2019; Sichel et al., 2022). The *C. jejuni 1104* gene overlaps by 64 bp with and is directly downstream of the M23 peptidase gene *1105*. This genomic arrangement of having a bactofilin gene overlapping with and downstream of an M23 peptidase domain-containing gene is conserved in most curved and helical organisms (Figure 1B; Sycuro et al., 2010; Sichel and Salama, 2020).

#### 3.1.3. M23 peptidase domain-containing protein CJJ81176_1105

A previous study by our group described a reduction of cell curvature resulting from a frameshift mutation in *1105* isolated by a passage on CFW (Frirdich et al., 2017). Another group identified *1105* in a visual screen for cell shape mutants in a Tn mutant library, with a Tn insertion in *1105* resulting in a decrease in cell curvature (Esson et al., 2017). The *1105* gene product has 35.0% identity/55.8% similarity to *H. pylori* 26695 Csd1 (HP1543) and 30.0% identity/50.0% similarity to *H. pylori* 26695 Csd2 (HP1544). Csd1 is an endopeptidase, and Csd2 is a homolog of Csd1 but has an inactive catalytic site (An et al., 2016). Csd2 and the Csd7 scaffolding protein are required for Csd1 stability (Yang et al., 2019). Neither an inactive homolog of 1105/Csd1 (similar to Csd2) nor a homolog of the scaffolding protein Csd7 have been identified in *C. jejuni*. Csd2 homologs have only been found in *H. pylori* and *H. hepaticus* (Sycuro et al., 2010).

Conserved domain analysis of 1105 (300 amino acids) by jackhmmr identified a transmembrane region (amino acids 28-47), a coiled-coil domain (amino acids 54-102), and an M23 peptidase domain (PF01551; amino acids 162-258) containing the conserved metalloprotease HxxxD and HxH motifs (Figure 1; Supplementary Figure S2). Interestingly, Csd1 and Csd2 do not have the coiled-coil domain found in *C. jejuni* 1105 and conserved among other *Campylobacter* spp. and members of the LmdC clade of M23 peptidases to which 1105, Csd1, and Csd2 belong (Figueroa-Cuilan et al., 2021). Coiled-coil domains are ubiquitous and highly versatile repetitive peptide motifs consisting of two or more α-helices wound around each other into a supercoil with a hydrophobic core (Burkhard et al., 2001). They can have various biological functions. For example, they are widely involved in oligomerization, many have been proposed to act as molecular spacers separating functional domains, and they have been shown to act as molecular rulers confining enzymatic activity to certain subcellular locations and less commonly act as a scaffold in protein complexes (Truebestein and Leonard, 2016).

#### 3.1.4. M23 peptidase domain-containing protein CJJ81176_1228

The *1228* gene product and its role in determining the degree of *C. jejuni* cell curvature were first described as part of a previous study (Stahl et al., 2016). 1228 shows 45% identity/65% similarity to the *H. pylori* 26695 Csd3 DD-carboxypeptidase and DD-endopeptidase (HP0506; Sycuro et al., 2010). *H. pylori* Csd3 has a predicted N-terminal transmembrane region and was crystallized without this transmembrane region in its latent, inactive form (An et al., 2015). The structure can be divided into three domains: domain 1 which occludes the active site of the M23 (LytM) domain, domain 2 of unknown function, and the C-terminal M23 (LytM) domain containing the catalytic Zn^2+^ binding site making up domain 3 (An et al., 2015; Supplementary Figure S3). The *C. jejuni* 1228 protein (386 amino acids) is predicted by jackhmmr to have a signal peptide (amino acids 1-19), the Csd3 N-terminal inhibitory domain (PF18059; Csd3 domain 1; amino acids 23-105), and the M23 peptidase domain (PF01551; Csd3 domain 3; amino acids 238-333) with the conserved active site HxxxD and HxH motifs (Figure 1A; Supplementary Figure S3). Activation of Csd3 was suggested to occur by removal of the inhibitory domain 1 from the active site by either autoproteolysis or cleavage by another endopeptidase (An et al., 2015). This may be similar to 1228. Proteins with a similar architecture are unique to members of the ε-Proteobacteria.

### 3.2. The 0166, 1104, 1105, and 1228 proteins are required for maintaining the degree of *Campylobacter jejuni* cell curvature

**Figure 2:**
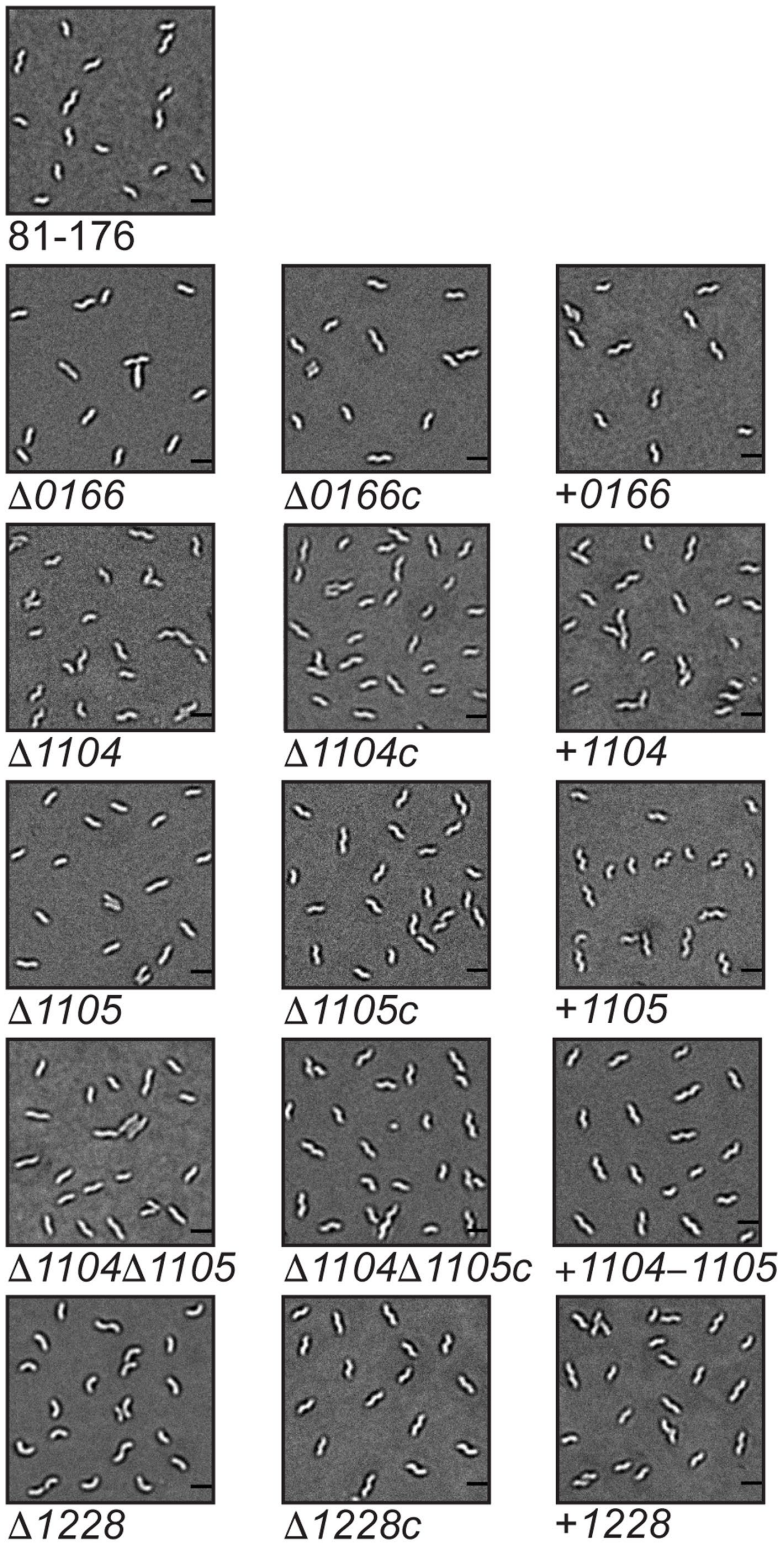
Morphology of *Campylobacter jejuni 0166*, *1104*, *1105*, *1104*–*1105*, and *1228* mutant, complement, and overexpressor strains. DIC microscope images of the helical *Campylobacter jejuni* 81-176 strain, the ∆*0166*, ∆*1104*, ∆*1105*, ∆*1104*, ∆*1105*, and ∆*1228* mutant strains and corresponding complemented (∆*0166c*, ∆*1104c*, ∆*1105c*, ∆*1104*, ∆*1105c*, and ∆1228c) and overexpression strains (denoted + *0166*, + *1104*, + *1105*, + *1104*–*1105*, and + *1228*). The scale bar indicates 2 μm.

The methods used to examine the morphology of strains are described here followed by the results and discussion of the analyses in separate sections organized by gene name. To determine the roles of the *0166*, *1104*, *1105*, and *1228* gene products in *C. jejuni* morphology, deletion mutants were constructed in each of the genes using a non-polar kanamycin resistance (Km^R^) cassette (Menard et al., 1993; Figure 1B; Supplementary material). Since 1104 and 1105 may interact, a ∆*1104*∆*1105* double mutant was also generated to examine the effect of losing both genes. This mutant was constructed by deleting *1105* with a chloramphenicol resistance (Cm^R^) cassette in an ∆*1104* (Km^R^) background (Yao et al., 1993; Figure 1B; Supplementary material). The ∆*1105* (Km^R^) and ∆*1105* (Cm^R^) mutants had similar effects on morphology (Figure 2; data not shown). Complementing and overexpressing strains were constructed using pRRC (Karlyshev and Wren, 2005) or pRRA (Cameron and Gaynor, 2014) integrative vectors (Supplementary material). All mutants, complements, and overexpressing strains showed wild-type growth characteristics (data not shown). The cell morphologies of the ∆*0166*, ∆*1104*, ∆*1105*, ∆*1104*∆*1105*, and ∆*1228* mutants, complementing, and overexpressing strains were assessed by microscopy. Unlike ∆*pgp1* and ∆*pgp2* that were uniformly rod-shaped (Frirdich et al., 2012, 2014), the ∆*0166*, ∆*1104*, ∆*1105*, and ∆*1228* mutants all retained a degree of cell curvature but differed in the amount of curvature [Figure 2 (DIC) and S4 (TEM)]. The roles of *1105* (Frirdich et al., 2017) and *1228* (Stahl et al., 2017) in maintaining cell curvature were previously described microscopically and followed up by quantification in this study.

To compare the subtle curvature changes between strains, morphological parameters were assessed by both MicrobeJ (Ducret et al., 2016) and Celltool (Pincus and Theriot, 2007; Sycuro et al., 2010; Ha et al., 2016; Liu et al., 2016) software programs. These two independent yet complementary analysis tools were used to maximize the description of curvature changes as they have different outputs: MicrobeJ displays mean values, while CellTool provides graphical representations of curvature changes. For quantification, DIC images of mid-exponential phase cells were taken from multiple fields of view totaling approximately 300 cells/strain and converted to binary images. Each with its own algorithms, the MicrobeJ and Celltool analysis programs detect the contour of each cell in the binary image. Then, they generate and record these as polygonal outlines along with their corresponding central axis. The central axis (referred to as the medial axis in MicrobeJ) runs from pole to pole through the middle of the cell and can be used to determine the morphological attributes of the cell.

We used MicrobeJ to quantify differences in cell length, width, and angularity (Table 1). The length is defined as the length of the central axis, the width is the mean width along the central axis, and angularity is defined as the mean curvature (the reciprocal of the radius of a circle tangent to a curve) values measured along the central axis. Within a wild-type population, variation in cell length accounts for most of the morphological variation in that population as shown by the larger standard deviation associated with the length, in contrast to the very small standard deviation in width and angularity. The variation in cell length in a population is expected since the population of cells will be at different stages of growth and division.

**Table 1:** MicrobeJ quantitative analysis of cell morphology parameters of *Campylobacter jejuni* wild-type 81-176, ∆*0166*, ∆*1104*, ∆*1105*, ∆*1104*∆*1105*, and ∆*1228* mutant strains, complemented strains ∆*0166*c, ∆*1104*c, ∆*1105*c, ∆*1104*, ∆*1105*c, and ∆*1228*c, and overexpression strains 81-176 + *0166*, 81–176 + *1104*, 81–176 + *1105*, 81–176 + *1104*–*1105*, and 81-176 + *1228*.^1^

*Campylobacter jejuni* strain	Sample size	Length (μm)	Width (μ∂m)	Angularity (1/μm)
81-176	302	1.53 ± 0.32	0.39 ± 0.02	0.19 ± 0.06
∆*0166*	271	1.58 ± 0.32	0.41 ± 0.02^****^	0.10 ± 0.05^****^
∆*0166c*	301	1.58 ± 0.32	0.40 ± 0.03^****^	0.19 ± 0.07
81-176 + *0166*	271	1.46 ± 0.31	0.40 ± 0.03^**^	0.21 ± 0.08
∆*1104*	300	1.56 ± 0.29	0.39 ± 0.02	0.19 ± 0.07
∆*1104c*	301	1.55 ± 0.30	0.39 ± 0.02	0.11 ± 0.05^****^
81-176 + *1104*	302	1.65 ± 0.31^****^	0.37 ± 0.02^****^	0.10 ± 0.05^****^
∆*1105*	316	1.62 ± 0.35^*^	0.38 ± 0.02^**^	0.12 ± 0.05^****^
∆*1105c*	326	1.44 ± 0.32^*^	0.35 ± 0.03^****^	0.19 ± 0.07
81-176 + *1105*	312	1.42 ± 0.29^***^	0.37 ± 0.03^****^	0.30 ± 0.10^****^
∆*1104*, ∆*1105*	300	1.69 ± 0.32^****^	0.40 ± 0.03 ^**^	0.11 ± 0.04 ^****^
∆*1104*, ∆*1105*c	296	1.49 ± 0.30	0.39 ± 0.02	0.20 ± 0.07
81-176 + *1104*–*1105*	321	1.48 ± 0.29	0.38 ± 0.02^**^	0.16 ± 0.07 ^****^
∆*1228*	310	1.66 ± 0.32^****^	0.42 ± 0.03^****^	0.27 ± 0.08^****^
∆*1228c*	277	1.55 ± 0.30	0.39 ± 0.02	0.20 ± 0.06
81-176 + *1228*	298	1.54 ± 0.30	0.39 ± 0.02	0.18 ± 0.06

1 Numbers represent mean values and standard deviations. Measurements were taken along the central axis of the cell with the width being the mean width along the central axis and angularity defined as the mean of the curvature values taken along the central axis. Values that are statistically significant in comparison to the wild-type 81–176 strain are indicated by an asterisk with ^*^, ^**^, ^***^, or ^****^ indicating *p* < 0.05, *p* < 0.01, *p* < 0.001, and *p* < 0.0001, respectively, and determined using Dunn’s correction for multiple comparisons after a Kruskal–Wallis test.

**Figure 3:**
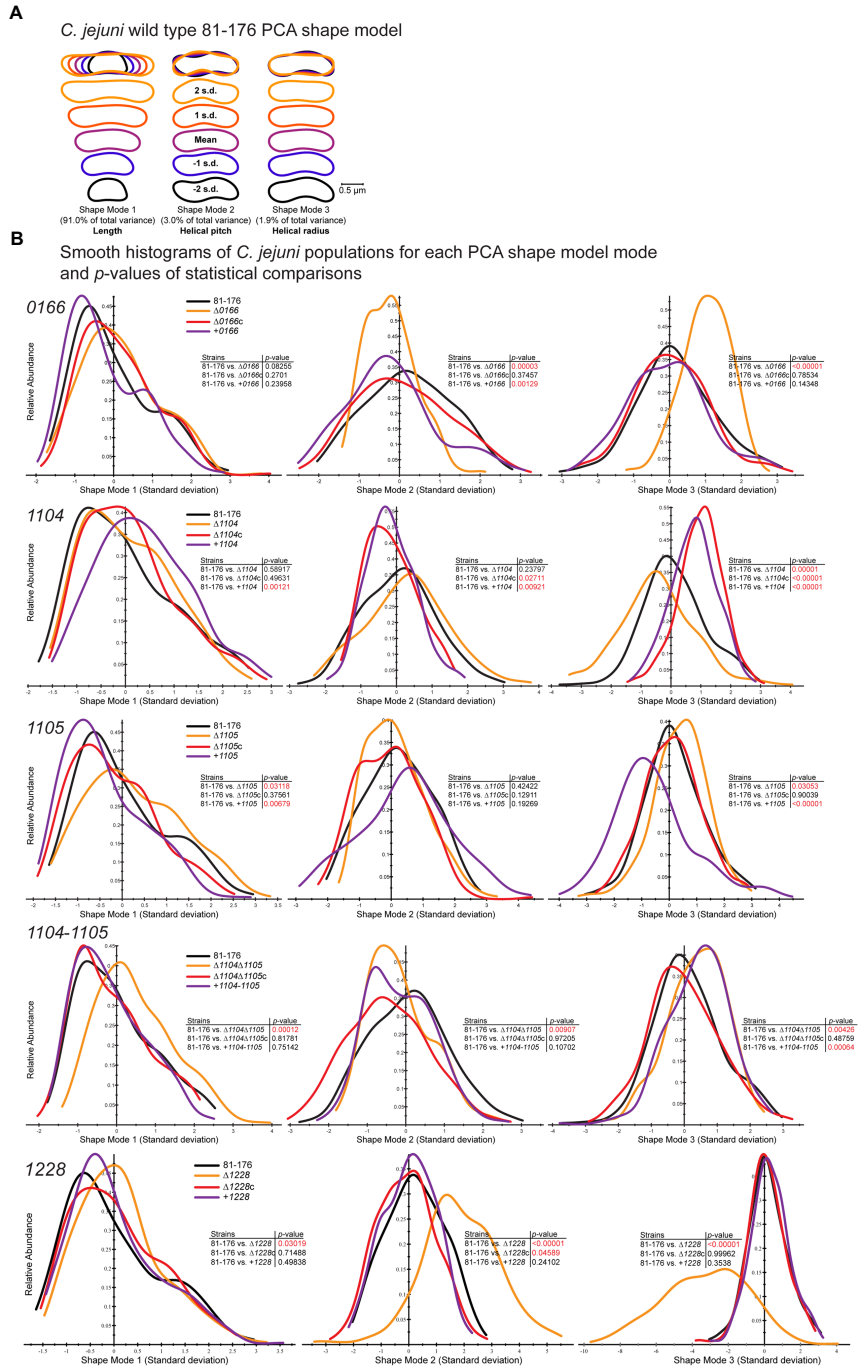
CellTool PCA analysis of population morphology for *Campylobacter jejuni 0166*, *1104*, *1105*, *1104*–*1105*, and *1228* mutant, complement, and overexpressor strains. *Campylobacter jejuni* strains grown to the mid-exponential phase were imaged by DIC microscopy. The images were converted to binary, and cell contours were extracted and aligned. **(A)** A *C. jejun*i wild-type shape model was generated from aligned contours by PCA analysis to explain 94.9% of the population variation with the principal component shape modes 1–3. The mean shape mode ±2 standard deviations (s.d.) are shown. Shape mode 1 represents length, shape mode 2 helical pitch, and shape mode 3 helical radius. CellTool was then used to align the contours of mutant and associated complemented and overexpression strain population morphologies to the wild-type shape model. **(B)** Measurements representing the normalized standard deviations from each wild-type shape mode relative to abundance are represented as a histogram for each mutant (∆*0166*, ∆*1104*, ∆*1105*, *∆1104*, *∆1105*, and *∆1228*), complement (∆*0166c*, ∆*1104c*, ∆*1105c*, *∆1104*, *∆1105c*, and ∆*12285*), and overexpressor (+*0166*, +*1104*, +*1105*, +*1104*–*1105*, and + *1228*). The analyses are grouped by gene: *0166*, *1104*, *1105*, *1104*–*1105*, and *1228*. The *p*-values comparing each strain to the wild type were determined by the Kolmogorov–Smirnov statistics and are shown as an inset to each graph. Values in red highlight statistically significant comparisons with *p*-values of < 0.05.

CellTool was used to compare differences in population morphology using the principal component analysis (PCA; Figure 3), as well as differences in the distributions of axis length (length of the central axis), normalized axis curvature (a measure of the normalized curvature of the central axis), and side curvature (a measure of the normalized curvatures along the contour itself excluding the poles; Supplementary Figure S5). As mentioned earlier, the curvature is the reciprocal of the radius of a circle tangent to a curve at any point. CellTool normalizes curvature to the arc length represented by the radius of curvature. The average of the absolute values of the pointwise curvature is computed over a specified range and multiplied by the contour length over the same range. The CellTool analysis was carried out as previously described (Pincus and Theriot, 2007; Sycuro et al., 2010; Ha et al., 2016; Liu et al., 2016). For PCA, an average *C. jejuni* wild-type cell shape was generated by aligning the contours of the wild-type population. PCA was performed to generate a shape model based on principal components called shape modes that describe changes in the population. Three shape modes describe 95% of the morphological variation within the wild-type *C. jejuni* 81-176 population (Figure 3A). Shape mode 1 describes the variation in cell length and represents 91% of the variance within the population, as seen with the MicrobeJ analysis. Shape mode 2 describes the variation in helical pitch ranging from a large (stretched out helix; 2 s.d. in shape mode 2; Figure 3) to a smaller pitch (compressed helix; − 2 s.d. in shape mode 2; Figure 3) and represents 3.0% of the variance. Shape mode 3 describes the variation in the helical radius ranging from straight (2 s.d. in shape mode 3; Figure 3) to curved (−2 s.d. in shape mode 3; Figure 3) and represents 1.9% of the variance. Contours of other strains were then aligned using the wild-type PCA shape model as a reference.

#### 3.2.1. Morphological analysis of ∆*0166*, complement, and overexpressor strains

The ∆*0166* mutant displayed a pleomorphic morphology, with some straight cells and some cells with varying decreased curvature (Figure 2; Supplementary Figure S4) as indicated by the reduction in mean angularity (Table 1) and the shifted distribution of normalized axis curvature and side curvature (Supplementary Figure S5). The PCA analysis (Figure 3) showed no variation in cell length (shape mode 1) but statistically significant variation in helical pitch (shape mode 2) and helical radius (shape mode 3). The curvature defects were complemented by wild-type morphology in all morphological attributes (Table 1; Figures 2, 3; Supplementary Figure S5). Overexpression of *0166* had a subtle effect on morphology, displaying a slight change only in the distribution of the helical pitch (Figure 3, shape mode 2).

#### 3.2.2. Morphological analysis of ∆*1104*, ∆*1105*, ∆*1104*∆*1105*, and corresponding complement and overexpressor strains

The ∆*1104* mutant morphology was similar to that of the wild type both visually (Figure 2; Supplementary Figure S4) and in all quantitative parameters (Table 1; Figure 3; Supplementary Figure S5) examined except for PCA shape mode 3 (helical radius), which showed a very slight increase in curvature that was not reflected in the MicrobeJ angularity values (Table 1). Complementation and *1104* overexpression resulted in a decrease in angularity (Table 1), with an increase in helical pitch (Figure 3, shape mode 2) and a decrease in helical radius (Figure 3, shape mode 3). The overexpressing strain also showed an increase in cell length and a decrease in width (Table 1; Figure 3, shape mode 1). The changes in the morphology of the complement are likely due to higher than wild-type levels of *1104* resulting from the expression at the non-native locus from the CmR promoter, as these changes were further accentuated in the overexpressing strain. Therefore, a dose of 1104 appears to affect morphology.

The ∆*1105* mutant morphology showed a decrease in curvature that was consistent across the population and not pleomorphic like in ∆*0166* (Figure 2; Supplementary Figure S4). In comparison to the wild type, there was a reduction in mean angularity and normalized axis curvature and side curvature (Table 1; Supplementary Figure S5) like that of ∆*1104c* and 81-176 + *1104*. The complement restored the changes in angularity, axis curvature, and side curvature but showed a larger decrease in width (Table 1; Supplementary Figure S5). Overexpression of *1105* resulted in the opposite effect as the deletion, with an increase in angularity (Table 1) which was also evident from the DIC images (Figure 2). The width of the overexpressing strain was more similar to that of the mutant (Table 1). PCA analysis demonstrated changes in shape mode 3 (helical radius) in the mutant and overexpressing strains, with the mutant having less of a helical radius (less curvature) than the wild type, and the overexpressor having a greater helical radius (more curvature; Figure 3), supporting the changes in angularity values. There were no changes in shape mode 2 (helical pitch). The mean length values (Table 1) and distribution of length (Figure 3, shape mode 1) showed an increase in length in the mutant, a slight decrease with the complemented strain, and a more pronounced decrease with the overexpressing strain. Thus, like 1104, a dose of 1105 also appears to affect morphology.

A ∆*1104*∆*1105* double mutant displayed a loss of cell curvature similar to the ∆*1105* mutant (Figure 2) with a reduction in angularity (Table 1), normalized axis curvature and side curvature (Supplementary Figure S5), an increase in cell radius (Figure 3, shape mode 3), and a lengthening in helical pitch (Figure 3, shape mode 2). The length of ∆*1104*∆*1105* cells was longer than that of a single ∆*1105* mutant (Table 1; Figure 3, shape mode 1). Complementation of ∆*1104*∆*1105* with *1104–1105* restored all wild-type morphological characteristics. The *1104–1105* overexpressing strain showed a decrease in angularity in comparison to the wild type that was not as pronounced as the ∆*1104*∆*1105* mutant (the *1104* overexpressing strain also showed a decrease in angularity while the *1105* overexpressing strain displayed an increase over wild-type levels; Table 1). The helical radius (Figure 3, shape mode 3) of the *1104–1105* overexpressing strain was similar to that of the ∆*1104*∆*1105* mutant and the *1104* overexpressing strain. Overall, the morphology of ∆*1104*∆*1105* mutant resembled that of ∆*1105*. Overexpression of *1104–1105* resulted in morphological characteristics similar to *1104* but not *1105* overexpression.

#### 3.2.3. Morphological analysis of ∆*1228*, complement, and overexpressor strains

The ∆*1228* mutant displayed variable curved rod morphologies primarily consisting of C- and S-shaped cells (Figure 2 and Supplementary Figure S4). Unlike ∆*0166* and ∆*1105* which had a reduction in angularity and curvature in comparison to the wild type, the angularity of ∆*1228* was increased compared to the wild type (Table 1), as was the normalized axis curvature and side curvature (Supplementary Figure S5). The ∆*1228* helical pitch (shape mode 2) and helical radius (shape mode 3) were both increased and had a broader distribution in comparison to the wild type (Figure 3). The ∆*1228* mutant also was longer and slightly wider than the wild type (Table 1; Figure 3, shape mode 1). The length and width were restored in the complemented strain (Table 1). The angularity (Table 1), helical radius (Figure 3, shape mode 3), and normalized curvature (Supplementary Figure S5) were restored in the complemented strain; however, the side curvature distribution was between that of the wild type and the mutant and not completely complemented (Supplementary Figure S5). The mean helical pitch (Figure 3, shape mode 2) was restored to the wild type but with a slightly altered distribution. The overexpressing strain was similar to the wild type (Table 1; Figure 3; Supplementary Figure S5) indicating that the lack of complementation with some parameters was not due to the overexpression but could be due to the expression from only the non-native promoter.

#### 3.2.4. Comparison of *Campylobacter jejuni* morphology to that of *Helicobacter pylori* homologs

A mutant in *H. pylori csd1* (*1105* homolog) showed a similar decrease in curvature to that of ∆*1105* (Sycuro et al., 2010). The ∆*csd1* mutant was very slightly wider than the wild type (Sycuro et al., 2010), while ∆*1105* was narrower (Table 1). *H. pylori* ∆*ccmA* (*1104* homolog) was less curved than *H. pylori csd1* (Sycuro et al., 2010), unlike ∆*1104* which was very similar to the wild type except for a very slight increase in helical radius (Table 1; Figure 3; Supplementary Figure S5). The morphology of mutants in the homologs of *C. jejuni 1228* in *H. pylori* (*csd3*/*hdpA*) varied depending on the strain of *H. pylori*. The *H. pylori* strain G27 *csd3* mutant displayed variable curved morphologies, with the majority of the cells being highly curved or C-shaped and a small amount having little to no curvature (Sycuro et al., 2010). These cells were slightly wider and shorter than the wild type. A mutant in the gene corresponding to the Csd3 homolog in *H. pylori* strain N6, ∆*hdpA*, was significantly wider and shorter than the wild type, forming stocky cells (Bonis et al., 2010). In addition, a portion of the ∆*hdpA* population displayed branched cells with additional poles. Note that the morphology of the *H. pylori* N6 strain shows very little curvature, unlike that of G27 (Bonis et al., 2010). The morphology of *C. jejuni* ∆*1228* consisted of cells with variable curvature, which as noted were primarily C- and S-shaped but with less curvature than those of ∆*csd3* (Table 1; Figure 3; Supplementary Figure S5; Sycuro et al., 2010). The ∆*1228* cells were longer than the wild type (Table 1) unlike ∆*csd3* (Sycuro et al., 2010) and ∆*hdpA* (Bonis et al., 2010) which were shorter. The widths of all the ∆*1228*, ∆*csd3*, and ∆*hdpA* mutants were greater than that of their corresponding wild type. Of note, the double mutant *∆pgp2∆1228* (described below) displayed some branched cells like ∆*hdpA* and cells with additional non-polar flagella as seen by TEM (data not shown).

### 3.3. Peptidoglycan analyses of ∆*0166*, ∆*1104*, ∆*1105*, and ∆*1228* reveal changes from the wild type and differences to the *Helicobacter pylori* homologs (where applicable)

As morphology is maintained by the PG layer, changes in the cell curvature are usually accompanied by changes in the muropeptide profile. PG was isolated from wild-type, mutant, complemented, and overexpressor strains to identify how 0166, 1104, 1105, and 1228 affect the muropeptide composition of *C. jejuni in vivo*. Muropeptide profiles were analyzed by HPLC as done previously (Frirdich et al., 2012, 2014, 2017, 2019; Ha et al., 2016; Table 2; Supplementary Figure S3, with the structures of the PG species identified shown for reference in Supplementary Figure S6). Changes described are in comparison to the wild type analyzed in parallel unless otherwise noted. Generally, only changes in muropeptide species that differed by 20% or more are discussed below. It is important to keep in mind when analyzing muropeptide profiles that even small changes in muropeptides present in small amounts, such as total pentapeptides in *C. jejuni*, have a high percent change, while larger changes in muropeptides present in larger amounts, such as total tetrapeptides, can have a low percent change.

**Table 2:** Summary of the muropeptide composition of *C. jejuni* wild-type 81-176, ∆*0166*, ∆*1104*, ∆*1105*, ∆*1104*, ∆*1105*, and ∆*1228* mutant strains, complemented strains ∆*0166*c, ∆*1104*c, ∆*1105*c, ∆*1104*, ∆*1105*c, and ∆*1228*c, and overexpression strains 81–176 + *0166*, 81–176 + *1104*, 81–176 + *1105*, 81–176 + *1104–1105*, and 81–176 + *1228*.^1^

			% Peak area in *Campylobacter jejuni* strains^2^															
Muropeptide	81-176 (a)	81-176 (b)^3^	81-176 (c)	81-176 (d)	81-176 (e)	∆*0166* (a)^4^	∆*0166c* (a)	81-176+*0166* (a)	∆*1104* (b)	*∆1104c* (b)	81-176 + *1104* (b)	∆*1105* (c)	∆*1105c* (d)	81-176 + *1105* (d)	∆*1104* ∆*1105* (e)	∆*1228* (c)	∆*1128c* (d)	81-176 + *1228* (d)
Monomers (Total)	41.6	44.0	40.9	41.2	42.2	44.1	39.9	40.3	42.0	41.9	41.7	37.5	40.6	43.1	39.0	**54.0** ^ * ^	41.0	41.0
Di	16.2	11.1	17.9	16.1	13.6	**9.7** ^ * ^	11.5 ^*^	12.5 ^*^	11.9	14.3	15.3 ^*^	16.4	13.3 ^*^	15.5	14.0	**10.7** ^ * ^	14.3	15.3
Tri	7.9	13.3	7.2	8.4	10.1	**12.2** ^ * ^	8.8	**9.6** ^ * ^	10.4 ^*^	10.2 ^*^	**8.9** ^ * ^	5.7 ^*^	10.0	8.9	**4.2** ^ * ^	**18.8** ^ * ^	8.5	7.7
Tetra	17.5	18.7	15.8	16.7	17.2	19.3	19.6	18.3	18.2	16.2	16.2	15.3	17.2	18.7	19.5	**24.5** ^ * ^	18.3	17.9
Penta	nd^5^	nd	nd	nd	nd	**2.9** ^ * ^	nd	nd	nd	nd	nd	nd	nd	nd	nd	nd	nd	nd
PentaGly5	nd	0.7	nd	nd	0.8	nd	nd	nd	0.5 ^*^	0.9	0.8	nd	nd	nd	nd	nd	nd	nd
Acetylated^6^	1.0	0.3	1.8	0.6	1.9	1.0	0.8	0.7	0.3	0.3	**0.5** ^ * ^	1.6	0.5	0.7	0.7	1.9	0.5	0.6
Dimers (Total)	50.3	48.0	50.4	49.5	48.8	50.7	50.3	51.3	49.6	48.9	48.2	52.6	49.8	48.5	51.1	42.2	49.5	49.2
TetraTri	16.8	16.3	15.8	15.7	16.0	12.4 ^*^	14.6	15.8	15.9	16.6	15.9	14.2	16.2	14.5	12.8	13.2	15.0	15.6
TetraTetra	33.1	30.4	33.1	33.1	32.0	30.2	35.1	35.0	32.7	30.9	30.7	36.8	32.8	33.2	37.6	27.7	33.8	33.0
TetraPenta	0.5	1.3	2.0	0.8	0.8	**8.0** ^ * ^	0.6	0.6	1.0	1.4	1.6 ^*^	1.6 ^*^	0.8	0.8	0.7	**1.3** ^ * ^	0.7	0.6 ^*^
Anhydro	12.4	9.5	12.0	12.0	11.8	8.8 ^*^	11.8	11.8	9.7	10.7	11.2	11.3	11.8	11.9	11.1	**7.3** ^ * ^	12.1	12.4
Acetylated^6^	1.7	0.0	2.6	0.3	3.2	1.6	0.3	1.8	nd	0.7 ^*^	0.0	2.1	0.2	0.3	nd	2.0	0.2	0.4
Trimers (Total)	8.1	7.1	8.6	9.3	7.8	**5.2** ^ * ^	9.8 ^*^	8.4	7.4	7.9	**8.7**	9.9	9.6	8.4	8.9	**3.8** ^ * ^	9.5	9.9
TetraTetraTri	1.2	0.8	1.3	1.2	1.0	**0.8** ^ * ^	1.2	1.1	0.9	1.0	0.9	1.4	1.3	1.0	1.0	**0.4** ^ * ^	1.2	1.3
TetraTetraTetra	6.8	6.3	7.4	8.1	6.8	**4.4** ^ * ^	8.5 ^*^	7.2	6.5	6.8	**7.7**	8.4	8.3	7.4	7.9	**3.4** ^ * ^	8.3	8.6
Dipeptides (Total)	16.2	11.1	17.9	16.1	13.6	**9.7** ^ * ^	11.5 ^*^	12.5 ^*^	11.9	14.3	**15.3** ^ * ^	16.4	13.3	15.5	14.0	**10.7** ^ * ^	14.3	15.3
Tripeptides (Total)	16.7	21.7	15.2	16.6	18.4	18.7	16.5	17.8	18.7	18.8	17.2 ^*^	13.3	18.6	16.4	**11.0** ^ * ^	**25.5** ^ * ^	16.4	16.0
Tetrapeptides (Total)	66.8	64.9	65.8	66.9	65.1	62.1	71.6	69.3	66.7	63.9	64.3	69.4	67.7	67.7	72.4	63.1	69.0	68.4
Pentapeptides (Total)	0.2	1.4	1.0	0.4	0.4	**6.9** ^ * ^	**0.3** ^ * ^	**0.3** ^ * ^	1.0 ^*^	1.6	1.6	0.8 ^*^	0.4	0.4	0.3 ^*^	**0.7** ^ * ^	0.3 ^*^	0.3 ^*^
Acetylated (Total) ^6^	1.9	0.3	3.1	0.8	3.5	1.7	0.9	1.6	0.3	0.7 ^*^	**0.5** ^ * ^	2.7	0.6	0.8	0.7	2.9	0.6	0.8
Anhydro chain ends (Total)	7.8	6.5	7.7	7.6	7.7	**5.3** ^ * ^	7.6	7.5	6.7	7.5	7.8 ^*^	7.5	7.6	7.4	7.4	**4.4** ^ * ^	7.7	7.9
Average chain length	12.9	15.4	12.9	13.2	13.0	**18.8** ^ * ^	13.2	13.4	15.0	13.4	12.8	13.3	13.2	13.6	13.5	**22.9** ^ * ^	13.0	12.7
Degree of cross-linkage	30.5	28.7	31.0	31.0	29.6	28.8	31.7	31.2	29.7	29.7	29.9	32.9	31.3	29.8	31.5	23.6 ^*^	31.1	31.2
% Peptides in cross-links	58.4	56.0	59.1	58.8	57.8	55.9	60.1	59.7	58.0	58.1	58.3	62.5	59.4	56.9	61.0	46.0 ^*^	59.0	59.0

1 Numbers represent the percent area of each muropeptide from Supplementary Table S3 calculated to give a total of 100%. Corresponding structures are shown in Supplementary Figure S5. Underlined values differ by 10% or more than the wild type analyzed in the corresponding sample set; values underlined and with an asterisk (^*^) differ by 20% or more; and values underlined with an asterisk and bolded differ by 30% or more.

2 The letter in parenthesis following the strain designation indicates the sample set. Samples in the same sample set will have the same letter designation, and a comparison between strains and wild type was carried out with the corresponding wild type from the same sample set.

3 Analysis of 81-176 (b) was carried out several years later than 81-176 (a), (c), (d), and (e). The differences in the ratio of di- to tri- in 81-176 (b) (which were consistent with other analyses of 81-176 carried out at the same time, data not shown) likely reflect changes in growth state due to differences in media composition. We have observed variations in results due to differences in batches of MH growth media. Changes in the ratio of di- to tri- also occur as *C. jejuni* age (Frirdich et al., 2019).

4 A subsequent analysis of the muropeptide profile of ∆*0166* determined the PentaGly5 levels to increase 4-fold in comparison to the wild type (data not shown). These data were not shown as they did not include an analysis of ∆*0166*c and 81-176 + 0166.

5 nd = not detected or not determined.

6 The values for the percentage of O-acetylated species do not represent the true level of O-acetylation in these strains, as most O-acetyl groups are lost in the standard procedure used in this study to reduce the muropeptides. These values were included to demonstrate the relative difference in O-acetylation between the samples, but actual comparisons between the samples were not made.

#### 3.3.1. Muropeptide profile of ∆*0166*, complement, and overexpressor strains

The ∆*0166* muropeptide profile displayed numerous significant changes compared to the wild type and was the only mutant muropeptide profile to show a relatively large increase in pentapeptides both in the monomeric and dimeric forms, with the largest increase occurring in the dimeric form (Table 2). The overall total dipeptides decreased and monomeric tripeptides increased. The TetraTri dimer levels were reduced which may be a result of the lack of processing of TetraPenta species. There was a decrease in overall levels of trimers but no change in the total amount of monomers and dimers. The amount of anhydro chain ends was reduced which was accompanied by a corresponding increase in average glycan strand length but no change in cross-linking. The complemented strain showed near complementation of most but not all of the muropeptide changes, such as the decrease in dipeptides. The number of trimers in the complement was more than the wild type, while that of the mutant was less than in the wild type. The *0166* overexpressing strain showed wild-type trimers as well as a decrease in dipeptides and an increase in monomeric tripeptides, with more tripeptides than in the complemented strain. Differences between the complement and overexpressing strain can result from the expression of *0166* from a non-native promoter. As such, this muropeptide analysis suggests that 0166 plays a role in processing pentapeptides. Both the *0166* deletion and *0166* overexpressing strains had increased the amounts of tripeptides and decreased dipeptides, indicating that the levels of 0166 in the cell could have an effect on Pgp1, which cleaves tri- to di-peptides. The decrease in the anhydro chain ends also suggests a possible effect on lytic transglycosylase activity, which is involved in generating anhydro sugars. There are four predicted lytic transglycosylases in *C. jejuni*: MltG, MltD, Slt, and RlpA. Interestingly, the bioinformatic STRING analysis indicated a potential interaction between 0166 and the membrane-bound murein lytic transglycosylase MltD.

#### 3.3.2. Muropeptide profile of ∆*1104*, ∆*1105*, ∆*1104*∆*1105*, and corresponding complement and overexpressor strains

There were similar changes in the muropeptide profiles of ∆*1104* and ∆*1105* (Table 2), with the changes accentuated in the ∆*1104*∆*1105* mutant, suggesting that these genes may function in a similar pathway. This is also supported by the STRING analysis and data on the *H. pylori* homologs, CcmA and Csd1, that show CcmA indirectly regulates Csd1 activity (Yang et al., 2019). Since 1104 does not have a peptidase domain, it likely affects muropeptide changes through interactions with the 1105 peptidase, enhancing and/or localizing 1105 activity. The most significant muropeptide changes observed in ∆*1104*, ∆*1105*, and ∆*1104*∆*1105* are a decrease in monomeric tripeptides, which was most pronounced in ∆*1104*∆*1105*. The complemented strain ∆*1105c* showed a small increase in monomeric tripeptides (complementing the decrease in the mutant) and a decrease in dipeptides. These changes were not seen in an *1105* overexpressing strain, which was similar to the wild type. This was intriguing considering that the morphology of the *1105* overexpressing strain was more curved than the wild type (described earlier in Section 3.2.2). Changes in the morphology are therefore not always reflected in the muropeptide analysis. The ∆*1104c* strain showed no complementation of the reduction of tripeptides and a slight increase in dipeptides. The increase in dipeptides and decrease in tripeptides observed in ∆*1104c* were more pronounced in the *1104* overexpressing strain, indicating that these changes are likely due to the overexpression of *1104*. These changes are reflected in changes in the morphology of both ∆*1104c* and the *1104* overexpressing strain, which were less curved that the wild type and the mutant.

Another group analyzed the muropeptide profile of ∆*1105* in their 81-176 background and reported a lack of changes in comparison to the wild type (Esson et al., 2017). That work presented the HPLC chromatogram of the muropeptide profile but did not report the percent peak area of each muropeptide species in the chromatogram in relation to the area of all the peaks identified. Quantitative analyses, as shown here, highlight the subtle changes in muropeptide species such as those in ∆*1105* (Table 2).

The muropeptide profiles of *H. pylori* ∆*csd1*, ∆*csd2* (both homologs of *C. jejuni 1105*), and ∆*ccmA* (*C. jejuni 1104* homolog) showed an increase in TetraPenta dimers and a decrease in tetrapeptide monomers, characteristic of a loss of endopeptidase activity required for the cleavage of TetraPenta cross-links (Sycuro et al., 2010). This was not seen with ∆*1104* and ∆*1105*. Potentially, another DD-endopeptidase is substituting for 1105 activity in its absence, such as Pgp3 which was shown to have both DD-endopeptidase and DD-carboxypeptidase activity against synthetic peptides (Min et al., 2020). The muropeptide profiles of *H. pylori* ∆*csd1*, ∆*csd2*, and ∆*ccmA* mutants all show an increase in cross-linking, which did not occur in *C. jejuni* ∆*1104* and ∆*1105*.

#### 3.3.3. Muropeptide profile of ∆*1228*, complement, and overexpressor strains

There were many significant changes in the muropeptide profile of ∆*1228* in comparison to the wild type (Table 2). There was an increase in the total amount of monomers, with a concomitant decrease in dimers and trimers. The overall total dipeptides and pentapeptides (although this was minor) decreased and tripeptides increased, with no change in overall tetrapeptides. However, both monomeric tri- and tetra-peptide levels increased. The muropeptide profile of ∆*1228* showed a decrease in anhydroMurNAc-containing chain ends with an increase in average glycan strand length, as well as a decrease in cross-linking in comparison to the wild type. To date, this is the only *C. jejuni* mutant observed to have a change in cross-linking. As with ∆*0166*, the decrease in anhydroMurNAc chain ends indicates a possible effect on one of the lytic transglycosylases (MltG, MltD, Slt, and RlpA). The bioinformatic STRING analysis showed a potential interaction between 1228 and MltG. STRING also showed a potential interaction between 1228 and the PG synthase PbpA, which has glycosyltransferase and transpeptidase domains and which could potentially explain the changes in cross-linking. As well, STRING showed a potential interaction with other proteins involved with PG biosynthesis: the M23 peptidase and putative hydrolase 1105 and the amidase AmiA (Frirdich et al., 2019). All changes in the deletion strain were complemented. No additional significant changes were detected in the complemented strain or the *1228* overexpressing strain.

Deletion of the *C. jejuni* 1228 homolog HdpA of *H. pylori* strain N6 resulted in a muropeptide profile with an increase in monomeric and dimeric pentapeptides and a reduction in tetrapeptides (Bonis et al., 2010), while in an *hdpA* overexpressing strain, there was a decrease in pentapeptide species and an increase in tetrapeptides and tripeptides with an increase in monomers and a decrease in dimers (Bonis et al., 2010). The profile of the *hdpA* overexpressing strain is similar to that of ∆*1228*. The muropeptide profiles of ∆*hdpA* and the *hdpA* overexpressing strain suggested that HdpA possesses DD-carboxypeptidase and DD-endopeptidase activity, which was confirmed *in vitro* with purified protein (Bonis et al., 2010). The *H. pylori csd3* gene is a homolog of *hdpA* in *H. pylori* G27. The muropeptide profile of a *csd3* mutant also indicated that this protein has DD-carboxypeptidase and DD-endopeptidase activity, although the muropeptide profile was slightly different from that of ∆*hdpA* (Bonis et al., 2010; Sycuro et al., 2010). Of note, in the muropeptide profiles of both *H. pylori hdpA* and *csd3* mutants, the glycan strand length, the number of anhydro chain ends, and the degree of cross-linking were unchanged, unlike in *∆1228*. This again highlights that PG hydrolase homologs, even in related bacteria, do not necessarily have the same effect on the muropeptide profile and role in PG biosynthesis.

### 3.4. The effects of multiple hydrolase deletions: Double and triple PG hydrolase mutant analyses show a complex interdependency between 0166, 1105, and 1228

**Figure 4:**
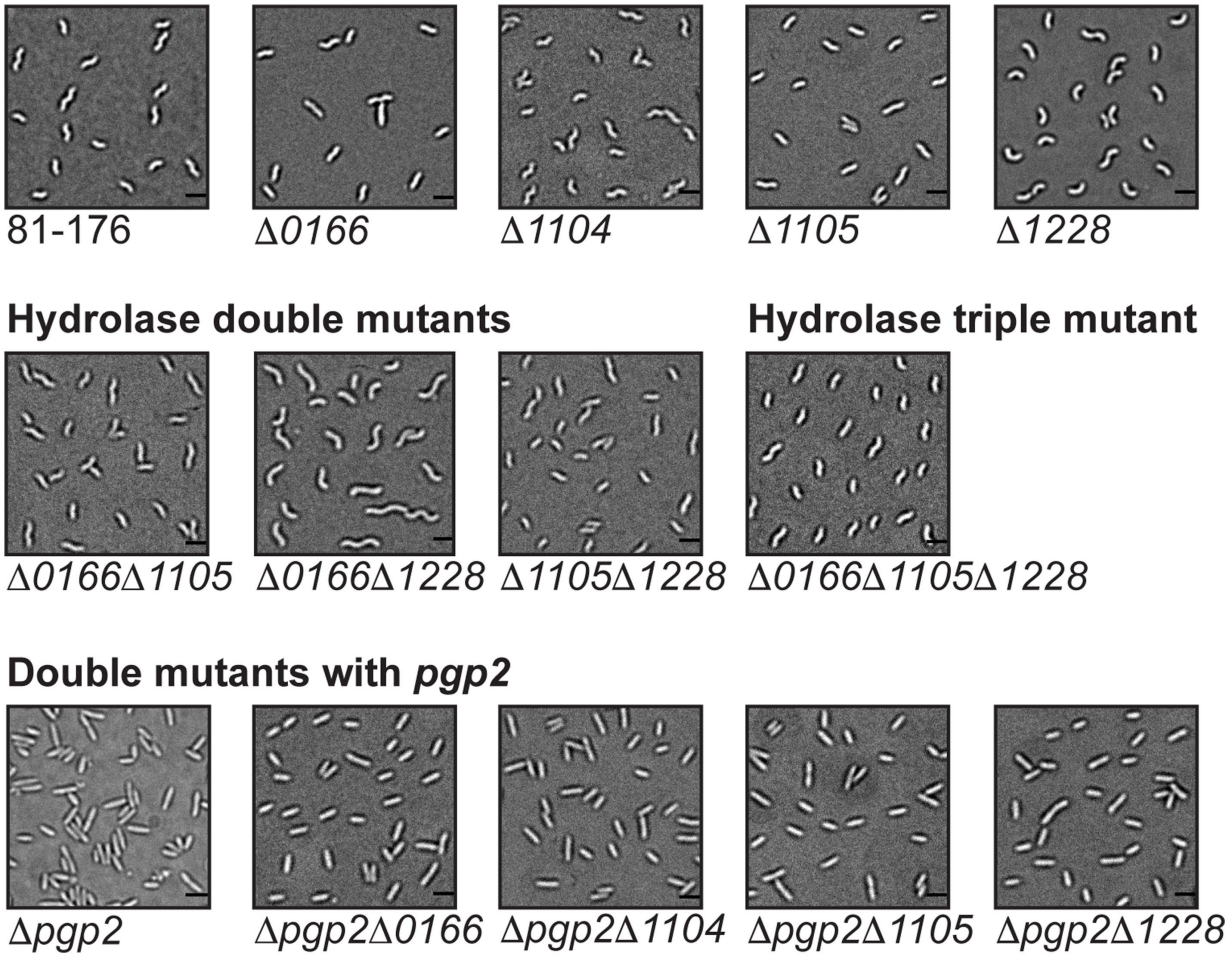
Mutant morphology of *C. jejuni* hydrolase double and triple mutants and double mutants with *pgp2*. DIC microscope images of hydrolase double mutants ∆*0166*, ∆*1105*, ∆*0166*, ∆*1228*, and ∆*1105*, ∆*1228* and the triple mutant ∆*0166*, ∆*1105*, ∆*1228*, as well as double mutants with *pgp2*, including images of the wild-type 81-176 strain and the single mutants for comparison. The scale bar indicates 2 μm.

Combinations of double mutants [∆*1104*∆*1105* (discussed earlier), ∆*0166*∆*1105*, ∆*0166*∆*1228*, and ∆*1105*∆*1228*], and a triple mutant (∆*0166*∆*1105*∆*1228*) were constructed to examine the relationships between these different peptidases and the effects of multiple hydrolase deletions. Each mutant was examined by DIC microscopy, and the muropeptide profiles were determined for the single mutants (Figure 4; Table 3; Supplementary Figure S4).

**Table 3:** Summary of the muropeptide composition of *0166*, *1105*, and *1228* double and triple mutants.^1^

	% Peak area in *Campylobacter jejuni* strains^2^				
Muropeptide	81-176 (f)	∆*0166* ∆*1105* (f)	∆*0166* ∆*1228* (f)	∆*1105* ∆*1228* (f)	∆*0166* ∆*1105* ∆*1228* (f)
Monomers (Total)	43.5	43.5	52.9 ^*^	40.7	45.1
Di	17.8	**11.5** ^ * ^	**10.5** ^ * ^	**10.2** ^ * ^	14.8
Tri	6.7	**12.5** ^ * ^	**19.8** ^ * ^	**10.2** ^ * ^	**9.7** ^ * ^
Tetra	18.3	16.1	22.1 ^*^	19.7	18.2
Penta	nd^3^	**1.5** ^ * ^	nd	nd	**0.8** ^ * ^
PentaGly5	0.6	**1.9** ^ * ^	0.5	0.6	**1.7** ^ * ^
Acetylated ^4^	1.0	1.8	1.6	2.3	1.1
Dimers (Total)	47.7	49.4	41.2	50.1	48.4
TetraTri	16.7	13.7	14.6	15.0	12.1 ^*^
TetraTetra	30.5	28.8	26.2	34.4	30.5
TetraPenta	0.4	**6.9** ^ * ^	0.4	**0.7** ^ * ^	**5.9** ^ * ^
Anhydro	13.5	**9.0** ^ * ^	**7.7** ^ * ^	10.2 ^*^	**9.1** ^ * ^
Acetylated ^4^	2.2	2.0	1.4	2.4	1.5
Trimers (Total)	8.8	7.1	**5.8** ^ * ^	9.2	**6.5** ^ * ^
TetraTetraTri	0.8	0.8	**0.5** ^ * ^	0.9	0.6 ^*^
TetraTetraTetra	8.0	6.3 ^*^	**5.3** ^ * ^	8.3	5.9 ^*^
Dipeptides (Total)	17.8	**11.5** ^ * ^	**10.5** ^ * ^	**10.2** ^ * ^	14.8
Tripeptides (Total)	15.4	**19.6** ^ * ^	**27.3** ^ * ^	18.0	15.9
Tetrapeptides (Total)	65.9	59.8	61.5	70.8	62.1
Pentapeptides (Total)	0.2	**4.9** ^ * ^	0.2	**0.3** ^ * ^	**3.7** ^ * ^
Acetylated (Total) ^4^	2.1	2.8	2.3	3.6	1.9
Anhydro chain ends (Total)	8.4	**5.4** ^ * ^	**4.7** ^ * ^	6.4 ^*^	**5.4** ^ * ^
Average chain length	12.0	**18.4** ^ * ^	**21.4** ^ * ^	**15.7** ^ * ^	**18.6** ^ * ^
Degree of cross-linkage	29.7	29.4	24.5	31.2	28.5
% Peptides in cross-links	56.5	56.5	47.1	59.3	54.9

1 Numbers represent the percent area of each muropeptide from Supplementary Table S4 calculated to give a total of 100%. Underlined values differ by 10% or more than the wild type analyzed in the corresponding sample set; values underlined and with an asterisk (^*^) differ by 20% or more; and values underlined with an asterisk and bolded differ by 30% or more.

2 The letter in parenthesis following the strain designation indicates the sample set. Samples in the same sample set will have the same letter designation, and a comparison between strains and wild type was carried out with the corresponding wild type from the same sample set.

3 nd = not detected or not determined.

4 The values for the percentage of O-acetylated species do not represent the true level of O-acetylation in these strains, as most O-acetyl groups are lost in the standard procedure used in this study to reduce the muropeptides. These values were included to demonstrate the relative difference in O-acetylation between the samples, but actual comparisons between the samples were not made.

The morphology of the ∆*0166*∆*1228* mutant resembled that of *∆1228* with a possible increase in variability in cell length (Figure 4). Following from this, the muropeptide profile of *∆0166∆1228* (Table 3) was identical to *∆1228* (Table 2), with no increase in pentapeptide characteristic of an *0166* deletion (Table 2). From these data, it would appear that *1228* is epistatic to *0166*.

Unlike *∆0166∆1228*, the morphology of the *∆1105∆1228* mutant displayed the relaxed curvature of ∆*1105* and not the C- and S-shaped cell characteristics of ∆*1228* (Figure 4). In contrast to ∆*1228*, the muropeptide profile of *∆1105∆1228* showed no change in the levels of monomers, dimers, and trimers, no increase in tetrapeptides, and an elevation in tripeptides that was at an intermediate level between the wild type and that of ∆*1228* (Tables 2, 3). An ∆*1105* mutant displayed decreased tripeptides in comparison to the wild type; as such, loss of *1105* may counteract the increase in tripeptides of ∆*1228*. Only the decrease in dipeptides and anhydro chain ends with a corresponding increase in average chain length of *∆1105∆1228* was similar to that of *∆1228*. As explained earlier, the effect on the anhydro chain ends and chain length would be due to an effect on lytic transglycosylase activity. From these results, it appears that 1105 may be required for 1228 function, be it either 1105 enzymatic activity or the presence of the protein itself.

The *∆0166∆1105* double mutant also displayed a relaxed curvature (Figure 4). The *∆0166∆1105* muropeptide profile was quite similar to that of ∆*0166* except for a slightly more pronounced increase in tripeptides and a less pronounced decrease in trimers (Tables 2, 3). The *∆1105* mutant did have a slight increase in trimers, which may have an antagonistic effect on the overall level of trimers in the double mutant.

The triple mutant *∆0166∆1105∆1228* had a similarly relaxed curvature to *∆0166∆1105* and *∆0166∆1228* (Figure 4). The *∆0166∆1105∆1228* mutant still retained a degree of curvature, indicating that the effects of these three genes on curvature are not additive. The morphology of the *∆0166∆1228* mutant resembled *∆1228*, but when ∆*1105* was also deleted to generate strain ∆*1105∆0166∆1228*, the morphology was similar to ∆*1105*. Based on morphology, *1105* would appear to be epistatic to *1228* which is epistatic to *0166*. This is more difficult to conclude based on the muropeptide profile. The muropeptide profile of the triple mutant had a less pronounced decrease in dipeptides and an increase in tripeptides than any of the double mutants, which was surprising, but a more pronounced increase in TetraTri dimers. The triple mutant did show an increase in pentapeptides similar to *∆0166∆1105* but not seen with *∆0166∆1228* and *∆1105∆1,228*.

In sum, the double and triple mutant muropeptide analyses revealed a complex interdependency between 0166, 1105, and 1228. Ongoing work to establish enzymatic activity and protein–protein interactions should provide increased insight into the relationships between these peptidases. Nonetheless, both the morphological characteristics shown in Figure 4, and the muropeptide analyses in Table 3 suggest that 1228 is required for the 0166 function, and to some extent, 0166 is required for the 1105 function and 1105 for 1228.

### 3.5. The LD-carboxypeptidase gene *pgp2* functions in a different pathway from *0166*, *1104*, *1105*, and *1228*

To determine the relationship among the *0166*, *1104*, *1105*, and *1228* genes involved in determining the degree of cell curvature and the *pgp2* gene required for generating helical cell shape (Frirdich et al., 2014), double mutants were constructed with *pgp2*. The ∆*pgp2*∆*0166*, ∆*pgp2*∆*1104*, ∆*pgp2*∆*1105*, and ∆*pgp2*∆*1228* double mutants were all rod-shaped similar to a ∆*pgp2* single mutant (Figure 4). However, despite their morphologies resembling that of ∆*pgp2*, the muropeptide profiles of the double mutants did show muropeptide differences characteristic of ∆*0166*, ∆*1104*, ∆*1105*, and ∆*1228* single mutants (Table 4; Supplementary Figure S5; described below) in addition to those characteristics of ∆*pgp2* (very little to no tripeptides and elevated tetrapeptides; Frirdich et al., 2014). This suggests that the proteins function independently and in different pathways, with Pgp2 activity not necessarily being required for their activity [unlike Pgp1 which requires Pgp2 to generate its substrate (Frirdich et al., 2014)].

**Table 4:** Summary of the muropeptide composition of double mutants with *pgp2* and *0166*, *1104*, *1105*, or *1228*.^1^

	% Peak area in *Campylobacter jejuni* strains^2^												
Muropeptide	∆*pgp2* ∆*0166* (f)	∆*pgp2* ∆*1104* (a)	∆*pgp2* ∆*1105* (a)	∆*pgp2* ∆*1228* (d)	∆*pgp2* ∆*0166* (f)	∆*pgp2* ∆*1104* (a)	∆*pgp2* ∆*1105* (a)	∆*pgp2* ∆*1228* (d)	∆*pgp2* ∆*0166* (f)	∆*pgp2* ∆*1104* (a)	∆*pgp2* ∆*1105* (a)	∆*pgp2* ∆*1228* (d)	∆*pgp2* (Frirdich et al., 2014)
Strain compared to:	**81–176 (f)**	**81–176 (a)**	**81–176 (a)**	**81–176 (d)**	**∆*0166***	**∆*1104***	***∆1105***	***∆1228***	***∆pgp2***	***∆pgp2***	***∆pgp2***	***∆pgp2***	
Monomers (Total)	49.3	41.4	39.5	**56.8** ^ * ^	49.3	41.4	39.5	56.8	49.3 ^*^	41.4	39.5	**56.8** ^ * ^	40.8
Di	**8.2** ^ * ^	**7.5** ^ * ^	**6.7** ^ * ^	**6.3** ^ * ^	8.2	**7.5** ^ * ^	**6.7** ^ * ^	**6.3** ^ * ^	8.2	7.5	6.7 ^*^	**6.3** ^ * ^	9.1
Tri	**0.5** ^ * ^	**0.5** ^ * ^	**0.5** ^ * ^	**0.5** ^ * ^	**0.5** ^ * ^	**0.5** ^ * ^	**0.5** ^ * ^	**0.9** ^ * ^	**0.5** ^ * ^	**0.5** ^ * ^	**0.5** ^ * ^	**0.9** ^ * ^	nd
Tetra	**35.9** ^ * ^	**33.5** ^ * ^	**32.3** ^ * ^	**49.6** ^ * ^	**35.9** ^ * ^	**33.5** ^ * ^	**32.3** ^ * ^	**49.6** ^ * ^	35.9	33.5	32.3	**49.6** ^ * ^	31.7
Penta	**2.3** ^ * ^	nd^3^	nd	nd	**2.3**	nd	nd	nd	**2.3** ^ * ^	nd	nd	nd	nd
PentaGly5	**2.3** ^ * ^	nd	nd	nd	2.3	nd	nd	nd	2.3	nd	nd	nd	nd
Acetylated ^4^	2.1	1.0	1.4	1.6	2.1	1.0	1.4	1.6	2.1	1.0	1.4	1.6	1.5
Dimers (Total)	44.5	50.6	52.2	40.1	44.5	50.6	52.2	40.1	44.5	50.6	52.2	40.1	47.6
TetraTri	nd	nd	nd	nd	nd	nd	nd	nd	nd	nd	nd	nd	nd
TetraTetra	37.0^*^	**50.0** ^ * ^	**51.2** ^ * ^	39.5	37.0	**50.0**	**51.2**	**39.5** ^ * ^	37.0 ^*^	50.0	51.2	39.5	46.8
TetraPenta	**7.4** ^ * ^	0.6 ^*^	**0.9** ^ * ^	**0.5** ^ * ^	7.4	**0.6** ^ * ^	**0.9** ^ * ^	**0.5** ^ * ^	**7.4** ^ * ^	0.6 ^*^	0.9	**0.5** ^ * ^	0.8
Anhydro	**8.8** ^ * ^	10.7	10.7	**7.3** ^ * ^	8.8	10.7	10.7	7.3	8.8 ^*^	10.7	10.7	**7.3** ^ * ^	12.1
Acetylated ^4^	0.6	1.5	1.9	0.1	0.6	1.5	1.9	0.1	0.6	1.5	1.9	0.1	0.4
Trimers (Total)	6.2 ^*^	8.0	8.3	**3.1** ^ * ^	6.2	**8.0**	8.3	**3.1** ^ * ^	**6.2** ^ * ^	**8.0** ^ * ^	8.3 ^*^	**3.1** ^ * ^	11.6
TetraTetraTri	nd	nd	nd	nd	nd	nd	nd	nd	nd	nd	nd	nd	nd
TetraTetraTetra	6.2 ^*^	8.0	8.3 ^*^	**3.1** ^ * ^	**6.2** ^ * ^	8.0	8.3	3.1	**6.2** ^ * ^	**8.0** ^ * ^	8.3 ^*^	**3.1** ^ * ^	11.6
Dipeptides (Total)	**8.2** ^ * ^	**7.5** ^ * ^	**6.7** ^ * ^	**6.3** ^ * ^	8.2	**7.5** ^ * ^	**6.7** ^ * ^	**6.3** ^ * ^	8.2	7.5	6.7 ^*^	**6.3** ^ * ^	9.1
Tripeptides (Total)	**0.5** ^ * ^	**0.5** ^ * ^	**0.5** ^ * ^	**0.9** ^ * ^	**0.5** ^ * ^	**0.5** ^ * ^	**0.5** ^ * ^	**0.9** ^ * ^	**0.5** ^ * ^	**0.5** ^ * ^	**0.5** ^ * ^	**0.9** ^ * ^	0.0
Tetrapeptides (Total)	80.2 ^*^	**91.7** ^ * ^	**92.3** ^ * ^	**92.5** ^ * ^	**80.2**	**91.7**	**92.3**	**92.5** ^ * ^	80.2	91.7	92.3	92.5	90.5
Pentapeptides (Total)	**6.1** ^ * ^	**0.3** ^ * ^	**0.5** ^ * ^	0.3 ^*^	6.1	**0.3** ^ * ^	**0.5** ^ * ^	**0.3** ^ * ^	**6.1** ^ * ^	0.3 ^*^	0.5 ^*^	0.3 ^*^	0.4
Acetylated (Total) ^4^	2.4	1.8	2.3	1.6	2.4	1.8	2.3	1.6	2.4	1.8	2.3	1.6	1.8
Anhydro chain ends (Total)	**5.2** ^ * ^	7.0	7.1	**4.3** ^ * ^	5.2	7.0	7.1	4.3	**5.2** ^ * ^	7.0	7.1	**4.3** ^ * ^	8.0
Average chain length	**19.2** ^ * ^	14.2	14.0	**23.4** ^ * ^	19.2	14.2	14.0	23.4	**19.2** ^ * ^	14.2	14.0	**23.4** ^ * ^	12.5
Degree of cross-linkage	26.4	30.6	31.6	22.1 ^*^	26.4	30.6	31.6	22.1	26.4	30.6	31.6	**22.1** ^ * ^	31.6
% Peptides in cross-links	50.7	58.6	60.5	43.2 ^*^	50.7	58.6	60.5	43.2	50.7	58.6	60.5	43.2 ^*^	59.2

1 Numbers represent the percent area of each muropeptide from Supplementary Table S5 calculated to give a total of 100%. Underlined values differ by 10% or more than the wild type analyzed in the corresponding sample set; values underlined and with an asterisk (^*^) differ by 20% or more; and values underlined with an asterisk and bolded differ by 30% or more.

2 The letter in parenthesis following the strain designation indicates the sample set. Samples in the same sample set will have the same letter designation, and a comparison between strains and wild type was carried out with the corresponding wild type from the same sample set. The muropeptide profile of the wild type and mutants used for comparisons is found in Tables 2, 3.

3 nd = not detected or not determined.

4 The values for the percentage of O-acetylated species do not represent the true level of O-acetylation in these strains, as most O-acetyl groups are lost in the standard procedure used in this study to reduce the muropeptides. These values were included to demonstrate the relative difference in O-acetylation between the samples, but actual comparisons between the samples were not made.

The *∆pgp2∆0166* double mutant showed an increase in total pentapeptides, as well as a decrease in the anhydro chain ends and an increase in chain length characteristic of *∆0166*.

The muropeptide profile of the *∆pgp2∆1105* double mutant showed a further reduction in dipeptides in comparison to *∆pgp2*. This is a change not present in ∆*1105*. There was an increase in TetraTetraTetra trimers in comparison to the wild type as seen in ∆*1105* and a decrease in comparison to *∆pgp2*, with a decrease in total trimers in comparison to *∆pgp2*. The *∆pgp2∆1104* double mutant profile was similar to that of *∆pgp2∆1105* except that there was less of a reduction in dipeptides. A *H. pylori ∆csd1∆csd6* double mutant (homologous to *∆pgp2∆1105*) showed changes that were characteristic of each of the single mutants with elevated TetraPenta dimers similar to *∆csd1* and increased tetrapeptides and reduced tri- and di-peptides similar to *∆csd6* (Sycuro et al., 2013). In addition, the morphology of *∆csd1∆csd6* was intermediate to both single mutants, unlike that of *∆pgp2∆1105* which resembled ∆*pgp2*.

The *∆pgp2∆1228* mutant showed some muropeptide changes that were accentuated in the double mutant in comparison to the single mutants, most notably a decrease in dipeptides and an increase in tetrapeptides. Some changes noted were characteristic of ∆*1228*: an increase in monomers, a decrease in dimers and trimers, a reduction in the number of anhydro chain ends, an increase in chain length, and a decrease in cross-linking.

## 4. Conclusion

Several genes in the *C. jejuni* genome encode proteins with predicted functions in PG biosynthesis, with only a few to date having been characterized as having a role in *C. jejuni* bacterial morphogenesis: Pgp1 (Frirdich et al., 2012), Pgp2 (Frirdich et al., 2014), Pgp3 (Esson et al., 2017), and AmiA (Frirdich et al., 2019). These enzymes are involved in generating the helical shape of *C. jejuni* (Pgp1, Pgp2, and Pgp3) and/or the transition to the coccoid form in response to stress (Pgp1 and AmiA), both of which influence pathogenesis and/or pathogenic properties. AmiA also plays a role in cell separation during cell division (Frirdich et al., 2019). This study describes the identification of four additional *C. jejuni* genes with a role in morphology generation, *0166*, *1104*, *1105*, and *1228*, and determined the effect of these genes on morphology and PG muropeptide composition. The *1104* gene encodes a putative bactofilin, a cytoskeletal protein with reported roles in bacterial morphology maintenance in other bacteria (Ramos-Leon and Ramamurthi, 2022). The *0166*, *1105*, and *1228* genes encoding M23 domain-containing proteins are likely PG hydrolases with endopeptidase and/or carboxypeptidase activity. The enzymatic activity of these gene products is currently being established. The influence of deleting *0166*, *1104*, *1105*, and *1228* on *C. jejuni* pathogenic attributes will be described in a subsequent study.

Deletion of *0166*, *1104*, *1105*, and *1228* produced strains with varying curvature in comparison to the wild type: The ∆*1104* mutant had a very slight increase in cellular helical radius, ∆*1105* was less curved, ∆*0166* was less curved but pleomorphic with morphologies ranging from straight rods to slightly curved, and ∆*1228* displayed variable curved rod morphologies of C- and S-shaped cells. The expression of *1104* from a non-native promoter in the complemented and overexpressing strains resulted in a decrease in cell curvature more pronounced than in the *1104* mutant. In contrast, *1105* deletion resulted in reduced curvature and *1105* overexpression in increased curvature. Therefore, a dose of both 1104 and 1105 appears to play an important role in determining wild-type helical morphology.

Unlike with *C. jejuni ∆pgp1* and ∆*pgp2*, specific assignments of 0166, 1105, and 1228 hydrolase function from the mutant muropeptide profiles of ∆*0166*, ∆*1105*, and ∆*1228* were not possible. There are several potential explanations for this. (1) PG hydrolases can have more than one enzymatic function, such as *C. jejuni* Pgp3 which was shown to have endopeptidase and carboxypeptidase activity *in vitro* (Min et al., 2020). (2) Different hydrolases can have redundant functions and can substitute for each other. For example, in *E. coli*, there are seven proteins with DD-endopeptidase activity (Pazos et al., 2017; Vermassen et al., 2019). (3) Changes in the levels of the 0166, 1104, 1105, and 1228 proteins (through deletion or overexpression) could affect other proteins in the PG biosynthetic complex by disruption of protein–protein interactions, resulting in numerous additional muropeptide changes. (4) PG hydrolases that further trim muropeptide species generated by the loss or overexpression of a hydrolase could also complicate the interpretation of the muropeptide profiles. The *in vitro* enzyme activities of 0166, 1105, and 1228 and the identification of their protein interaction partners will help establish the concrete roles these proteins play in *C. jejuni* PG biosynthesis and how they interact with each other and/or other PG biosynthetic enzymes such as the PG synthases, lytic transglycosylases, and the amidase AmiA.

Like *C. jejuni*, *Helicobacter pylori* is a member of the ε-Proteobacteria and has a helical morphology. *H. pylori* has homologs of Pgp1 (Csd4) and Pgp2 (Csd6) that have similar enzymatic functions and roles in helical shape generation as Pgp1 and Pgp2, respectively (Frirdich et al., 2012, 2014; Sycuro et al., 2012, 2013). However, we have observed that increases in Pgp1 levels also affect helical morphology which was not seen in *H. pylori* (Frirdich et al., 2012). Deletions in the *H. pylori* homologs for *1104* (*ccmA*), *1105* (*csd1*), and *1228* (*csd3*; Sycuro et al., 2010) had different morphological effects (except for *csd1*) and muropeptide changes compared to the *C. jejuni* gene products (note that an *H. pylori 0166* homolog has not been studied). It was suggested that trimming of PG cross-links enables wild-type helical cell curvature and twisting in *H. pylori*, as mutants in *ccmA*, *csd1*, and *csd3* with curved rods but not helical morphologies had increased levels of cross-linking (Sycuro et al., 2010). In contrast, deletions in *0166*, *1104*, and *1105* had no effect on total cross-linking, while a *1228* mutant had decreased levels of cross-linking. Cross-linking may still play a role in generating *C. jejuni* helical cell morphology, but these data suggest that it may be mechanistically different than in *H. pylori*.

Collectively, this study provides novel and detailed morphology and muropeptide analyses for four new *C. jejuni* proteins involved in PG modification. In addition, it also highlights that, despite their similar helical morphology and the presence of several homologous proteins, some of which have similar activities, the morphogenesis programs of *C. jejuni* and *H. pylori* have striking differences. These include differences in effects on the muropeptide profiles of homologous enzymes, in the complement of morphogenesis proteins (i.e., *H. pylori* has several proteins lacking homologs in *C. jejuni*, such as Csd2, Csd5, and Csd7), and possibly how they generate the helical shape. These similarities and differences will continue to be discovered as our understanding of how helical shape is generated in *C. jejuni* and *H. pylori* evolves. In addition, this study emphasizes the importance of studying the PG biosynthetic program in related but distinct bacterial pathogens.

## Data availability statement

The original contributions presented in the study are included in the article/Supplementary material, further inquiries can be directed to the corresponding authors.

## Author contributions

EF conceived and designed the study. EF and JV constructed the strains used in the study and carried out the morphological analysis. JB carried out the muropeptide analysis. Data analysis and interpretation was carried out by all authors. Funding was provided by EG and WV. EF wrote the manuscript which was revised in depth with EG. All authors contributed to the article and approved the submitted version.

## Funding

This study was funded by Canadian Institutes of Health Research grants 68981 and 461767 to EG. Work in WV’s laboratory was supported by the UKRI Strategic Priorities Fund (EP/T002778/1) and the BBSRC (BB/W005557/1).

## Conflict of interest

The authors declare that the research was conducted in the absence of any commercial or financial relationships that could be construed as a potential conflict of interest.

## Publisher’s note

All claims expressed in this article are solely those of the authors and do not necessarily represent those of their affiliated organizations, or those of the publisher, the editors and the reviewers. Any product that may be evaluated in this article, or claim that may be made by its manufacturer, is not guaranteed or endorsed by the publisher.
