# Supplementary material for: Multiple Campylobacter jejuni proteins affecting the peptidoglycan structure and the degree of helical cell curvature
Source: Front Microbiol. 2023 Apr 18;14:1162806. doi: 10.3389/fmicb.2023.1162806 (PMC10151779; doi:10.3389/fmicb.2023.1162806)
Supplement: Supplementary file 1 [file Data_Sheet_1.PDF]

Supplementary Material

Multiple *Campylobacter jejuni* proteins affecting peptidoglycan structure and the degree of helical cell curvature

Emilisa Frirdich*, Jenny Vermeulen, Jacob Biboy, Waldemar Vollmer, and Erin C. Gaynor*

* Correspondence: Emilisa Frirdich: emilisa@mail.ubc.ca; or Erin C. Gaynor: egaynor@mail.ubc.ca

List of Contents

1. Supplemental Materials & Methods

2. Supplemental Tables

Table S1. Bacterial strains and plasmids.
Table S2. Primers.
Table S3. Muropeptide composition of *C. jejuni* wild-type 81-176, Δ*0166,* Δ*1104,* Δ*1105,* Δ*1104*Δ*1105,* and Δ*1228* mutant strains, complemented strains Δ*0166*c, Δ*1104*c, Δ*1105*c, Δ*1104*Δ*1105*c, and Δ*1228*c and overexpression strains 81-176+*0166,* 81-176+*1104,* 81-176+*1105,* 81-176+*1104-1105,* and 81-176+*1228* summarized in Table 2.
Table S4. Muropeptide composition of *0166, 1105* and *1228* double and triple mutants summarized in Table 3.
Table S5. Muropeptide composition of double mutants with *pgp2* and *0166*, *1104*, *1105* or *1228* summarized in Table 4.

3. Supplemental Figures

Figure S1. 1104 alignment and domain identification.
Figure S2. 1105 alignment and domain identification.
Figure S3. 1228 alignment and domain identification.
Figure S4. Loss of curvature and pleiomorphic morphology of mutant strains.
Figure S5. CellTool analysis to examine differences in cell curvature in *C. jejuni 0166*, *1104*, *1105*, and *1228.*
Figure S6. Muropeptide structures.

4. References

1. Supplemental Materials & Methods

<u>Construction of *C. jejuni* mutant, complement and overexpressing strains</u>

Note that the Δ*1105* (Frirdich et al., 2017) and Δ*1228* (Stahl et al., 2016) mutants and their respective complementing strains were constructed as part of a previous study. For mutant construction, a portion of the gene of interest was replaced by the non-polar *aphA3* kanamycin (Km) resistance cassette from pUC18-Km (Menard et al., 1993) or the chloramphenicol (Cm) resistance cassette (*cat*). Genes were PCR amplified with iProof (Biorad) from *C. jejuni* 81-176 genomic DNA using primers 0166-L1 and 0166-R1 for *0166* and 1104-L1 and 1104-R1 for *1104*. A polyA tag was added to the PCR product and it was ligated to a commercially available pGEM-T vector (Promega). The resulting constructs (pGEMT-*0166* and pGEMT-*1104*) were verified by PCR analysis and sequencing. Inverse PCR was performed on pGEMT-*0166* with primers 0166-IL1 (*KpnI*) and 0166-IR1 (*XbaI*) (deleting nucleotides 348-1214 of the 1374 bp *0166* gene), digested with *KpnI* and *XbaI* and the product was ligated to the *aphA3* Km resistance cassette digested out of pUC18-Km with *KpnI* and *XbaI*, forming plasmid pGEMT-*0166*Km. Inverse PCR was performed on pGEMT-*1104* with primers 1104-IL1(*KpnI*) and 1104-IR1(*XbaI*) (deleting nucleotides 172-323 of the 396 bp *1104* gene), digested with *KpnI* and *XbaI* and the product was ligated to the *aphA3* Km resistance cassette digested out of pUC18-Km with *KpnI* and *XbaI*, forming plasmid pGEMT-*1104*Km. For the construction of an *1104-1105* double mutant, an *1105* mutant marked with Cm was constructed to delete *1105* in an Δ*1104* (Km) background. To generate an *1105* mutant with the Cm resistance cassette, inverse PCR was performed on pGEMT-*1105* (Frirdich et al., 2017) with primers 1105-IL1 and 1105-IRK (deleting nucleotides 325-682 of the 903 bp *1105* gene) and the product was ligated to a *cat* cassette digested out of pRY109 (Yao et al., 1993) with *KpnI* to form plasmid pGEMT-*1105*Cm. For each construct, correct insertion of the cassette in the same orientation as the gene was verified by PCR and restriction enzyme analysis. *C. jejuni* 81-176 was naturally transformed with pGEMT-*0166*Km and pGEMT-*1104*Km and mutants were selected on MH-TV containing Km. Mutants were designated Δ*0166* and Δ*1104* and verified by PCR analysis. A double mutant in Δ*1104* and Δ*1105* was constructed by deleting Δ*1105* in the Δ*1104* strain with pGEMT-*1105Cm*. The *Δ1104* mutant strain was transformed with pGEMT-*1105*Cm and mutants designated Δ*1104*Δ*1105* were selected on MH-TV containing Km and Cm. PCR analysis was used to verify deletion of *1105* and the original *1104* deletion.

For complementation and overexpression, the *0166* and *1104* genes were PCR amplified from *C. jejuni* 81-176 genomic DNA with primers 0166-L2 (*XbaI*) and 0166-R2 (*MfeI*) for 0166, and 1104-L2 (*XbaI*) and 1104-R2 (*MfeI*) for *1104*. The PCR product was digested with *XbaI* and *MfeI*, and cloned into the similarly digested pRRC integration vector (Karlyshev and Wren, 2005) in the same orientation as the antibiotic resistance cassette encoded by the vector. For complementation of Δ*1104*Δ*1105*, the *1104-1105* genes were PCR amplified with 1105-L2 (*XbaI*) and 1104-R3 (*MfeI*), digested with *XbaI* and *MfeI*, and cloned into the similarly digested pRRA integration vector (Cameron and Gaynor, 2014) in the same orientation as the antibiotic resistance cassette encoded by the vector. All constructs (pRRC-*0166*, pRRC-*1104*, pRRC-*1105* (Frirdich et al., 2017), pRRC-*1228* (pEF48; (Stahl et al., 2016)) and pRRA-*1104-1105*) were verified by PCR and sequencing and inserted by natural transformation into the respective mutant strains for complementation or *C. jejuni* wild type 81-176 for overexpression. Transformants were selected on MH-TV plates containing Cm for pRRC and Apr for pRRA.

Complemented strains were designated: Δ*0166-c*, Δ*1104-c,* Δ*1105-c* (Frirdich et al., 2017)*,* Δ*1104*Δ*1105-c,* and Δ*1228-c* (Stahl et al., 2016) and overexpressor strains: 81-176+*0166,* 81-176+*1104,* 81-176+*1105*, 81-176+*1104-1105* and 81-176+*1228*. Single insertions into the rRNA spacer region were verified by PCR with primers ak233, ak234, ak235 (Karlyshev and Wren, 2005) and ak237 for pRRC and apr-1 for pRRA.

2. Supplemental Tables

Table S1. Bacterial strains and plasmids

Strain or Plasmid	Genotype, serotype or description	Reference or Source
C. jejuni		
81-176	Wild type isolated from a diarrheic patient	(Korlath et al., 1985)
Δ*0166*	81-176 *0166*::*aphA3*; Km^R	This study
Δ*1104*	81-176 *1104*::*aphA3*; Km^R	This study
Δ*1105*	81-176 *1105*::*aphA3*; Km^R	(Frirdich et al., 2017)
Δ*1105*Cm	81-176 *1105*::*cat*; Cm^R	This study
Δ*1104*Δ*1105*	81-176 *1104*::*aphA3 1105*::*cat*; Km^RCm^R	This study
Δ*1228*	81-176 *1228*::*aphA3*; Km^R	(Stahl et al., 2016)
Δ*0166-c*	81-176 *0166*::*aphA3 rrn::0166*; Km^R Cm^R	This study
Δ*1104-c*	81-176 *1104*::*aphA3 rrn::1104*; Km^R Cm^R	This study
Δ*1105-c*	81-176 *1105*::*aphA3 rrn::1105;* Km^R Cm^R	(Frirdich et al., 2017)
Δ*1104*Δ*1105-c*	81-176 *1104*::*aphA3 1105*::*cat rrn::1104-1105*; Km^RCm^R Apr^R	This study
Δ*1228-c*	81-176 *1228*::*aphA3 rrn::1228*; Km^R Cm^R	(Stahl et al., 2016)
81-176+*0166*	81-176 *rrn::0166* (from pRRC-*0166*); Cm^R	This study
81-176+*1104*	81-176 *rrn::1104* (from pRRC-*1104*); Cm^R	This study
81-176+*1105*	81-176 *rrn::1105* (from pRRC-*1105*); Cm^R	This study
81-176+*1104-1105*	81-176 *rrn::1104-1105*; Apr^R	This study
81-176+*1228*	81-176 *rrn::1228* (from pEF48); Cm^R	This study
E. coli		
DH5α	F^-, φ80d *deoR lacZ*Δ*M15 endA1 recA1 hsdR17*(r_K-m_K+) *supE44 thi-1 gyrA96 relA1* Δ*(lacZYA-argF) U169*	Invitrogen
**Plasmids**		
pGEM-T	PCR cloning vector; Ap^R	Promega
pUC18-K2	Source of non-polar *aphA3* cassette; Ap^RKm^R	(Menard et al., 1993)
pRY109	Source of *cat* cassette; Ap^RCm^R	(Yao et al., 1993)
pRRC	*C. jejuni* rRNA spacer integration vector; Cm^R	(Karlyshev and Wren, 2005)
pRRA	*C. jejuni* rRNA spacer integration vector; Apr^R	(Cameron and Gaynor, 2014)
pGEMT-*0166*	pGEM-T ligated to the *0166* gene amplified with primers 0166-L1 and 0166-R1; Ap^R	This study

pGEMT-*0166*Km	pGEMT-*0166* with the *0166* gene disrupted with the *aphA3* cassette; Ap^R Km^R	This study
pGEMT-*1104*	pGEM-T ligated to the *1104* gene amplified with primers 1104-L1 and 1104-R1; Ap^R	This study
pGEMT-*1104*Km	pGEMT-*1104* with the *1104* gene disrupted with the *aphA3* cassette; Ap^R Km^R	This study
pGEMT-*1105*	pGEM-T ligated to the *1105* gene amplified with primers 1105-L1 and 1105-R1; Ap^R	(Frirdich et al., 2017)
pGEM-T-*1105*Km	pGEMT-*1105* with the *1105* gene disrupted with the *aphA3* cassette; Ap^R Km^R	(Frirdich et al., 2017)
pGEM-T-*1105C*m	pGEMT-*1105* with the *1105* gene disrupted with the *cat* cassette; Ap^R Cm^R	This study
pRRC-*0166*	pRRC derivative coding for *0166* amplified with 0166-L2 and 0166-R2; Cm^R	This study
pRRC-*1104*	pRRC derivative coding for *1104* amplified with 1104-L2 and 1104-R2; Cm^R	This study
pRRC-*1105*	pRRC derivative coding for 1105; Cm^R	(Frirdich et al., 2017)
pRRA-*1104-1105*	pRRA derivative coding for 1104-1105 amplified with 1105-L2 and 1104-R3; Apr^R	This study
pEF48	pRRC derivative coding for *1228*; Cm^R	(Stahl et al., 2016)

Table S2. Primers

Primer	Sequence 5′ to 3′	Restriction Site	Reference
ak233	GCAAGAGTTTTGCTTATGTTAGCAC		(Karlyshev and Wren, 2005)
ak234	GAAATGGGCAGAGTGTATTCTCCG		(Karlyshev and Wren, 2005)
ak235	GTGCGGATAATGTTGTTTCTG		(Karlyshev and Wren, 2005)
ak237	TCCTGAACTCTTCATGTCGATTG		(Karlyshev and Wren, 2005)
aphA3-2	CTATTTTTTGACTTACTGGGGA		(Frirdich et al., 2012)
apr-1	CGCTACGGAAGGAGCTGT		(Cameron and Gaynor, 2014)
0166-L1	GGTATGAGCAGAATTGCCA		This study
0166-R1	CTTTTACCTCTGCTTCATCAAG		This study
0166-IL1	acgggtaccGCTATCACCTCCAAAACAAAAG	*KpnI*	This study
0166-IR1	gcgtctagaGGAACAAGTGGTTTAGCCTTA	*XbaI*	This study
0166-L2	gcgtctagaggtatgagcagaattgcca	*XbaI*	This study
0166-R2	gcacaattgCTAAAGTAATTTCTATGGGTTCACC	*MfeI*	This study
1104-L1	AAGTGGAGTAGTGGAATTTTCA		This study
1104-R1	CTTATACTTTCAATCTCTTCGCC		This study
1104-IL1	acgggtaccTCCGATAACCACAATGCTTTC	*KpnI*	This study
1104-IR1	gcgtctagaCGGTCGCTTTAATGGAACTAG	*XbaI*	This study
1104-L2	gcgtctagaAGTGGAGTAGTGGAATTTTCA	*XbaI*	This study
1104-R2	gcacaattgGAAACTTGAGCGAGTAAATCTG	*MfeI*	This study
1104-L3	gcgtctagaTAATCAAGAAAGGAGAGTTCCATG	*XbaI*	This study
1104-R3	gcacaattgGGATTTTGTAGGTATTCATAAAATGAAGC	*MfeI*	This study
1105-L1	CTGCTAAGGCTATGCTTGAT		(Frirdich et al., 2017)
1105-R1	TGAGCGAGTAAATCTGCTTG		(Frirdich et al., 2017)
1105-IL1	acgggtaccTTCTACTTCTAAGCCCAAAGC	*KpnI*	(Frirdich et al., 2017)
1105-IRK	acgggtaccGGCAGTTGATAGGTTATAGCG	*KpnI*	This study
1105-IR1	gcgtctagaGGCAGTTGATAGGTTATAGCG	*XbaI*	(Frirdich et al., 2017)
1105-L2	gcgtctagaCTGCTAAGGCTATGCTTGAT	*XbaI*	(Frirdich et al., 2017)
1105-R2	gcacaattgTACCACTTAACTCTCCATCAAC	*MfeI*	(Frirdich et al., 2017)

Table S3. Muropeptide composition of *C. jejuni* wild-type 81-176, Δ*0166,* Δ*1104,* Δ*1105,* Δ*1104*Δ*1105,* and Δ*1228* mutant strains, complemented strains Δ*0166*c*,* Δ*1104*c*,* Δ*1105*c*,* Δ*1104*Δ*1105*c, and Δ*1228*c and overexpression strains 81-176+*0166,* 81-176+*1104,* 81-176+*1105,* 81-176+*1104-1105,* and 81-176+*1228* summarized in Table 2.

	**% Peak area in *C. jejuni* strains**[1]																	
**Muropeptide**[2]	**81-176 (a)**	**81-176 (b)**	**81-176 (c)**	**81-176 (d)**	**81-176 (e)**	**Δ*0166* (a)**	**Δ*0166c* (a)**	**81-176 +*0166* (a)**	**Δ*1104* (b)**	**Δ*1104c* (b)**	**81-176 +*1104* (b)**	**Δ*1105* (c)**	**Δ*1105c* (d)**	**81-176 +*1105* (d)**	**Δ*1104* Δ*1105* (e)**	**Δ*1228* (c)**	**Δ*1128c* (d)**	**81-176 +*1228* (d)**
Tri	6.9	13.0	6.4	7.5	8.8	11.0	7.9	8.7	10.1	9.8	8.5	5.1	9.1	8.0	4.1	17.3	7.6	6.9
TetraGly4	nd[3]	0.0	nd	nd	0.4	nd	nd	nd	0.7	0.2	0.2	nd	nd	nd	1.2	nd	nd	nd
Tetra	16.2	18.7	14.4	15.6	16.1	17.8	18.5	17.1	18.1	16.1	16.0	14.3	16.1	17.4	19.1	22.5	17.1	16.6
PentaGly5	nd	0.7	nd	nd	0.8	nd	nd	nd	0.5	0.9	0.8	nd	nd	nd	nd	nd	nd	nd
Di	15.0	11.1	15.3	14.8	13.0	nd	nd	nd	11.8	14.2	15.2	14.0	12.3	14.3	13.0	nd	nd	nd
Tri-Ac [4]	0.5	0.3	0.2	0.3	1.0	0.4	0.4	0.4	0.3	0.3	0.4	0.3	0.3	0.4	nd	0.3	0.3	0.3
Penta	nd	nd	nd	nd	nd	2.7	nd	nd	nd	nd	nd	nd	nd	nd	nd	nd	nd	nd
Tetra-Ac [4]	0.2	nd	0.2	nd	0.6	0.2	nd	0.2	nd	nd	0.2	nd	nd	0.1	nd	0.5	nd	0.1
Di-Ac	0.2	nd	1.3	0.7	0.2	0.2	0.3	nd	nd	nd	nd	1.3	0.2	0.2	0.7	1.0	0.1	0.1
TetraTri	11.6	13.7	10.1	11.2	10.5	9.0	10.6	11.5	13.4	13.3	12.5	9.9	11.7	10.5	9.8	10.2	10.8	10.9
TetraPentaGly5	0.4	1.3	1.9	0.8	0.8	2.7	0.6	0.6	1.0	1.4	1.6	1.5	0.8	0.8	0.7	1.2	0.6	0.6
TetraTetra	21.9	23.5	21.2	22.9	21.4	21.2	25.0	23.8	25.3	22.6	22.7	25.1	22.8	22.8	28.5	19.5	23.4	22.5
TetraAnh	nd	0.2	nd	nd	nd	nd	nd	nd	0.3	0.2	0.2	nd	nd	nd	nd	nd	nd	nd
TetraPenta	nd	nd	nd	nd	nd	4.8	nd	nd	nd	nd	nd	nd	nd	nd	nd	nd	nd	nd
TetraTri-Ac [4]	0.6	nd	0.9	0.2	1.5	0.5	0.3	0.6	nd	nd	nd	0.6	0.1	0.2	nd	0.6	0.1	0.3
TetraTetraTri	1.1	0.8	1.2	1.1	1.0	0.8	1.2	1.1	0.9	1.0	0.9	1.3	1.2	0.9	1.00	0.4	1.1	1.2
TetraTetra-Ac [4]	1.1	nd	1.5	0.1	1.6	0.9	nd	1.2	nd	0.7	nd	1.4	0.1	0.1	nd	1.3	0.1	0.1
TetraTetraTetra	2.1	2.7	2.1	3.1	2.5	1.5	3.4	2.3	2.8	2.4	3.0	2.6	3.2	3.0	3.3	1.2	3.2	3.4
TetraTriAnh I	1.2	1.1	1.2	1.1	1.1	0.8	0.8	0.8	1.0	1.3	1.4	1.0	1.1	1.0	0.8	0.6	0.9	1.1
TetraTriAnh II	2.4	1.5	2.0	2.1	2.7	1.3	2.2	2.1	1.5	1.9	1.9	1.8	2.2	1.9	1.8	1.0	2.2	2.3
TetraTetraAnh I	3.2	3.4	3.9	3.1	2.8	2.8	3.4	3.5	3.4	3.6	3.8	3.7	2.9	3.2	2.9	2.7	2.9	2.9
TetraTetraAnh II	4.9	3.5	4.0	4.9	5.2	3.3	4.8	4.8	3.8	3.8	4.0	4.1	4.8	5.1	5.3	2.6	5.3	5.3
TetraTetraTriAnh	nd	1.0	nd	nd	1.1	nd	nd	nd	1.0	1.3	1.4	nd	nd	nd	1.0	nd	nd	nd
TetraTetraTetraAnh	4.3	3.6	4.7	4.5	4.1	2.5	4.7	4.6	3.6	4.4	4.6	5.3	4.6	4.0	4.4	2.0	4.6	4.7
All known	93.96	100.0	92.4	93.6	96.8	93.2	94.8	95.1	99.4	99.6	99.3	93.3	93.6	93.7	97.6	94.1	93.6	93.5

[1] The number in parenthesis following the strain designation indicates the sample set. Samples in the same sample set will have the same letters.

[2] The order of muropeptide species represents the order in which they elute by HPLC.

[3] nd = not detected.

[4] The values for the percentage of *O*-acetylated species do not represent the true level of *O*-acetylation in these strains, as most *O*-acetyl groups are lost in the standard procedure used in this study to reduce the muropeptides. These values were included to demonstrate the relative difference in *O*-acetylation between the samples, but actual comparisons between the samples were not made.

Table S4. Muropeptide composition of *0166, 1105* and *1228* double and triple mutants summarized in Table 3.

	% Peak area in *C. jejuni* strains[1]				
**Muropeptide[2]**	**81-176 (f)**	**Δ*0166* Δ*1105* (f)**	**Δ*0166* Δ*1228* (f)**	**Δ*1105* Δ*1228* (f)**	**Δ*0166* Δ*1105* Δ*1228* (f)**
Tri	5.8	10.9	17.7	8.5	8.7
Tetra	16.1	14.8	20.2	18.4	16.6
PentaGly5	0.5	1.8	0.4	0.5	1.6
Di	16.2	10.6	9.9	9.6	14.0
Tri-Ac [3]	0.3	0.9	1.0	1.3	0.4
Penta	nd[4]	1.4	nd	nd	0.7
Tetra-Ac [3]	0.6	0.5	0.5	0.6	0.6
Di-Ac	nd	0.3	0.0	0.3	nd
TetraTri	9.7	9.2	11.0	9.9	8.3
TetraPentaGly5	0.4	2.2	0.4	0.7	2.2
TetraTetra	19.0	20.7	18.8	25.6	21.9
TetraPenta	nd	4.3	nd	nd	3.4
TetraTri-Ac [3]	1.8	1.8	1.0	2.0	0.9
TetraTetraTri	0.8	0.8	0.5	0.9	0.5
TetraTetra-Ac [3]	0.2	0.6	0.2	0.4	0.5
TetraTetraTetra	2.9	3.4	2.7	4.4	3.3
TetraTriAnh I	1.2	0.7	1.5	0.6	0.7
TetraTriAnh II	2.6	1.7	0.2	2.0	1.5
TetraTetraAnh I	3.0	2.3	2.0	2.6	2.6
TetraTetraAnh II	5.5	3.8	3.6	4.6	3.9
TetraTetraTetraAnh	4.3	2.6	2.3	3.6	2.3
All known	91.2	94.9	93.9	96.6	94.8

[1] The letter in parenthesis following the strain designation indicates the sample set. Samples in the same sample set will have the same letters.
[2] The order of muropeptide species represents the order in which they elute by HPLC.
[3] The values for the percentage of *O*-acetylated species do not represent the true level of *O*-acetylation in these strains, as most *O*-acetyl groups are lost in the standard procedure used in this study to reduce the muropeptides. These values were included to demonstrate the relative difference in *O*-acetylation between the samples, but actual comparisons between the samples were not made.
[4] nd = not detected.

Table S5. Muropeptide composition of double mutants with *pgp2* and *0166*, *1104*, *1105* or *1228* summarized in Table 4.

	% Peak area in *C. jejuni* strains[1]			
**Muropeptide**[2]	**Δ*pgp2* Δ*0166* (f)**	**Δ*pgp2* Δ*1104* (a)**	**Δ*pgp2* Δ*1105* (a)**	**Δ*pgp2* Δ*1228* (d)**
Tri	nd[3]	nd	nd	nd
Tetra	33.4	31.5	29.9	45.3
PentaGly5	2.2	nd	nd	nd
Di	7.6	6.9	5.9	5.7
Tri-Ac [4]	0.5	0.5	0.5	0.8
Penta	2.3	nd	nd	nd
Tetra-Ac [4]	1.2	0.3	0.4	0.5
Di-Ac	0.3	0.2	0.4	0.1
TetraTri	nd	nd	nd	nd
TetraPentaGly5	1.9	0.6	0.9	0.5
TetraTetra	26.7	35.9	36.2	29.6
TetraPenta	5.2	nd	nd	nd
TetraTri-Ac [4]	nd	nd	nd	nd
TetraTetraTri	nd	nd	nd	nd
TetraTetra-Ac [4]	0.5	1.5	1.8	0.1
TetraTetraTetra	3.6	2.8	2.8	1.2
TetraTriAnh I	nd	nd	nd	nd
TetraTriAnh II	nd	nd	nd	nd
TetraTetraAnh I	3.4	3.9	4.0	2.7
TetraTetraAnh II	5.0	6.3	6.0	4.1
TetraTetraTetraAnh	2.4	4.8	5.0	5.8
All known	96.5	95.1	93.6	92.2

[1] The letter in parenthesis following the strain designation indicates the sample set. Samples in the same sample set will have the same letters.
[2] The order of muropeptide species represents the order in which they elute by HPLC.
[3] nd = not detected.
[4] The values for the percentage of *O*-acetylated species do not represent the true level of *O*-acetylation in these strains, as most *O*-acetyl groups are lost in the standard procedure used in this study to reduce the muropeptides. These values were included to demonstrate the relative difference in *O*-acetylation between the samples, but actual comparisons between the samples were not made.

3. Supplemental Figures

```
BacA          --MFSKQAKSNNKAPARIEPLPTPMAATPAEPARRAPPKVASLLSADLTIEGGVTGEGEL      58
1104          MAIFNKGGISPTS-----------------------SSSSETTVISSGARIEGKFYFASML      38
CcmA          MAIFDNNNKSAN-----------------------AKTGPATIIAQGTKIKGELHLDYHL      37
                :*.:   * .                       :    ::::: .  *:* .     *

BacA          QIDGVVKGDVRV-GRLTVGETGHVEGSVYAEAVEVRGRVVGAITSKQVRLYGTSYVDGDI      117
1104          HVDGELSGIIHSESIVVIGKNGNLKGELQADKIVVNGYFEGQLEANSLEILAGGVVNGDI      98
CcmA          HVDGELEGVVHSKSTVVIGQTGSVVGEIFTNKLVVSGKFTGTVEAEVVEIMPLGHLDGKI      97
              ::** :.* ::  . :.:*:.* : *.: :: : * * . * : :: :.:   . ::*.*

BacA          THEQLAMETGAFFQGRSLKFQRPAPAPSQPAPHPEHLAIAKSAG      161
1104          STQKISIENGGRFNGTSKIKEDTIKLI-ENN-NEE---------      131
CcmA          SSQELVVERKGILIGETRPKNIQGGALLINE-QEKKI--ENK--      136
```

Figure S1. 1104 alignment and domain identification. Omega O (1.2.4) multiple sequence alignment of *C. jejuni* 81-176 1104 (CJJ81176_1104; 131 amino acids), *H. pylori* 26695 CcmA (HP1542; 136 amino acids), and the well studied bactofilin *Caulobacter crescentus* CB15 BacA [CC_1873; 161 amino acids; (Shi et al., 2015; Vasa et al., 2015)]. The bactofilin domain is shown in blue as identified by jackhmmr. The conserved potential membrane targeting motif (Deng et al., 2019) is shown in green. An asterisk (*) indicates a position with a fully conserved residue, a colon (:) conservation between groups of strongly similar properties, and a period (.) conservation between groups of weakly similar properties.

```
Csd2      -MPQNQLVITIIDESGSKQLKFSKNLKRN---LIISVVIFLLIVGLGVGFLKFLIAKMDT      56
1105      -MVKNKFTITITDINGSRHFYLNQIIKKIVFYTIAFIVLFLVFSGFYI---KYLDSKLSD      56
Csd1      MFLDRRLIVMVTDSKGSRYINVHILFRQIGLYALLSVVGSLLFLGISL---LVLNQEIKN      57
           : ..:: : : * .**: : .   :::     :  :*  *:: *: :     *  ::.

Csd2      MTAERNAVLRDFRDLYQKNYTLTKEIKNKREELFIVGQKIRTIESLIEVKRGANGGVH--      114
1105      ISEKREELLKKSKELELSNSQMQKSIEEKTQQYAAIEDKIASFEEALGLEVE----NNLT      112
Csd1      IDKQHALITKEFEKKKETNEKLSLQMDEFLDDLQLSGERINDLEEVVGVNRPEEEKEEGN      117
          :  ::  : :. ..   .*  :  .:.:  ::     ::*  :*. : ::       .

Csd2      LYDEVDLDNLNLAQKHLALMLIPNGMPIKTYSAIK-PTKERNHPIKKIKGVESGIDFIAP      173
1105      ISARLDNLQLTNEQQLGILGQIPNGWPIENK-GITGNFGWREHPLLKRREFHPGIDLRAE      171
Csd1      FSSRLDVAGITGLQKSFIMRLIPNDYPLESYRRVSAAFNKRIHPILHVLHNHTGLDLSTA      177
          :  .:*   :.  *:   :  ***. *::.   :.     * **: :    . *:*: :
                                                            HXXXD

Csd2      LNTPVYASADGIVDFVKTNSNVGYGNLVRIEHAFGFSSIYTHL-DHVNVQPKSFIQKGQL      232
1105      IGTPIYAPASGVVEFSGYS-DNGYSYNVILLHNFGFKTVFAHMMRKEVVKAGQFVSKGQL      230
Csd1      INTPVYASASGVVGLASKGWNGGYGNLIKVFHPFGFKTYYAHL-NKIVVKTGEFVKKGQL      236
          :.**:** *.*:* :   . : **.  : : * ***.: ::*:  :  *:  .*:.****

Csd2      IGYSGKSGNSGGEKLHYEVRFLGKILDAQKFLAWDLDHFQSALEENKFIEWKNLFWVLED      292
1105      IGYSGNTGLSTGPHLHYEVRFINKTLEPLYFLNLQRKNMNDFFNQERRVPWQSLIKAVSA      290
Csd1      IGYSGNTGMSTGPHLHYEVRFLDQPINPMSFTKWNMKDFEEVFNKERSIRWQSLITIINR      296
          *****::* * * :*******:.: ::   *   : ..::. ::::: : *:.*:  :.
                       HXH

Csd2      IVQLQEHVDKDALISQ          308
1105      QHPALAQKQQ------          300
Csd1      LMQKQDQRLSSLKAQK          312
                :  .
```

Figure S2. 1105 alignment and domain identification. Omega O (1.2.4) multiple sequence alignment of *C. jejuni* 81-176 1105 (CJJ81176_1105; 300 amino acids), *H. pylori* 26695 Csd1 (HP1543; 312 amino acids), and *H. pylori* 26695 Csd2 (HP1544; 308 amino acids). The transmembrane helix is shown in orange, the coiled coil region in yellow, and the M23 (LytM) domain in blue that contains the catalytic site, as identified by jackhmmr. The residues boxed in red belong to the characteristic HxxxD and HxH motifs involved in metalloprotease activity with yellow highlighted resides indicating the conserved M23 catalytic site residues and residues highlighted in blue indicate the missing active site residues in Csd2. Csd2 is an inactive homolog of Csd1. The M23 (LytM) domains determined by structural analysis of Csd1$_{125\text{-}312}$ and Csd2$_{121\text{-}308}$ are underlined and the helical domains highlighted in grey (An et al., 2015). Secondary structure analysis of 1105 using SABLE identified a similar helical domain as those in Csd1 and Csd2. An asterisk (*) indicates a position with a fully conserved residue, a colon (:) conservation between groups of strongly similar properties, and a period (.) conservation between groups of weakly similar properties.

```
1228      ----MKKLLLLFIFVV-----------------QSFAALSVEELTWDNGDTLLKFLQRNS      39
Csd3      MVFFHKKIIILNFIYSLMVAFLSHGVLLKADGMAKKQTLLVGERLVWDK-LTLLGFLEKNH      59
               **::* **: :                    :. : *  *.*.**:  *** **::*

1228      IPMSLYYGLDREDQELASDIAYKIKYQVLKDENNNIEQVLIPISDDLQIHIYKDKDGQYT      99
Csd3      IPQKLYYNLSSQDKELSAEIQSNVTYYTLRDANNTLIQALIPISQDLQIHIYKKG-EDYF      118
          ** .***.*. :*:**:::*  ::.* .*:* **.: *.*****:********.    :*

1228      LAFTPVSYQKEDRILHLTIKSSAYQDVYEESGSSTLARAMVRAFRGSINFRNIQKGDEVT      159
Csd3      LDFIPIIFTRKEKTLLLSLQTSPYQDIIKATNDPLLANQLMNAYKKSVPFKRLVKNDKIA      178
          * * *: : :::: * *::::* ***: : :..  **. ::.*:: *: *:.: *.*:::

1228      LYYEQKRRMGKLWGDINIKMAMVEINKSAREVFS-YNDIFYDRDGKELESFLLTKPVNYT      218
Csd3      IVYTRDYRVGQAFGQPTIKMAMVSSRSNQYYLFSHSNGHYYDSKAQEVAGFLLETPVKYT      238
          : * :. *:*: :*: .******. ...    :**  *. :** ..:*: .*** .**:**

1228      RISSPFTTARYHPILKRYRAHLGIDYAAPTGTPVKSAGKGVVTFIGTKGGYGNVIQIKHD      278
Csd3      RISSPFSYGRFHPVLKVRRPHYGVDYAAKHGSLIHSASDGRVGFMGVKAGYGKVVEIHLN      298
          ******: .*:**:**  * * *:****  *: ::**..* * *:*.*.***:*::*: :
                             HXXXD

1228      SGYMTLYAHLSRFA-KIKNGQKVNQGQVIAYVGSTGMSTGPHLHFGVYLNNKAINPASVV      337
Csd3      E-LRLVYAHMSAFANGLKKGSFVKKGQIIGRVGSTGLSTGPHLHFGVYKNSRPINPLGYI      357
          .    :***:* **  :*:*. *::**:*. *****:*********** *.: ***  . :
                                                HXH

1228      KIAKSELSGKAKENFKHIIAGYEQVVKEALVSNQPNPPKEEDFENYIEF      386
Csd3      RTAKSKLHGKQREVFLEKAQRSKQKLEELLKTHSFEK---NSFYLLEGF      403
          : ***:* ** :* *  .     :* ::* * ::. :    :.*     *
```

Figure S3. 1228 alignment and domain identification. Pairwise sequence alignment of *C. jejuni* 81-176 1228 (CJJ81176_1228; 386 amino acids) with the *H. pylori* 26695 Csd3 (HP0506; 403 amino acids) showing 39.9% identity and 58.2% similarity. Domains in 1228 were identified by jackhmmr. The locations of the domains in Csd3 were identified using the published crystal structure (An et al., 2015). The 1228 signal peptide is shown in red, the Csd3 transmembrane helix is shown in orange, domain 1 in green, domain 2 in purple, and the M23 (LytM) domain 3 in blue that contains the catalytic site. The residues boxed in red belong to the characteristic HxxxD and HxH motifs involved in metalloprotease activity with highlighted resides indicating the conserved residues. An asterisk (*) indicates a position with a fully conserved residue, a colon (:) conservation between groups of strongly similar properties, and a period (.) conservation between groups of weakly similar properties.

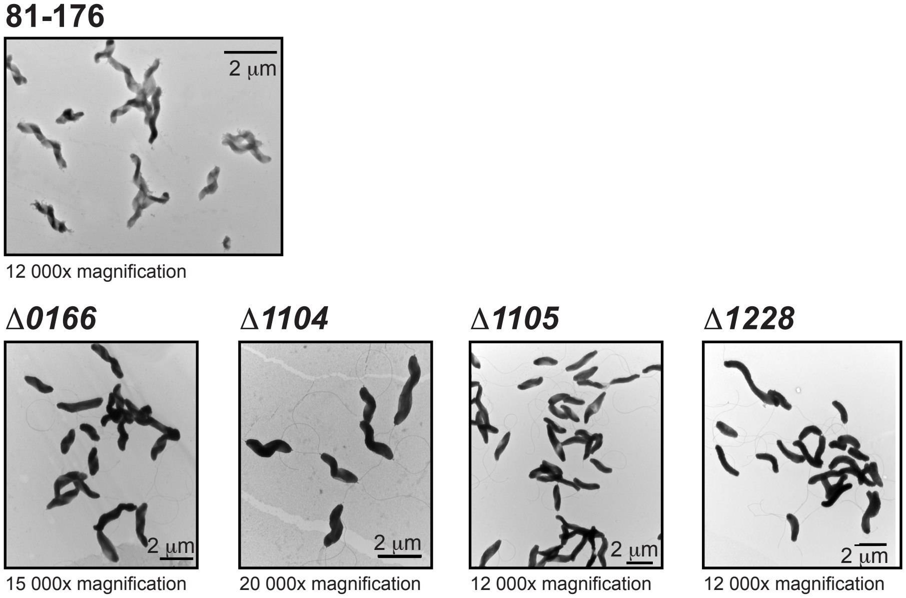

Figure S4. Loss of curvature and pleimorphic morphology of mutant strains. Negatively stained TEM images of *C. jejuni* wild type 81-176 and Δ*0166*, Δ*1104*, Δ*1105* and Δ*1228* mutant strains.

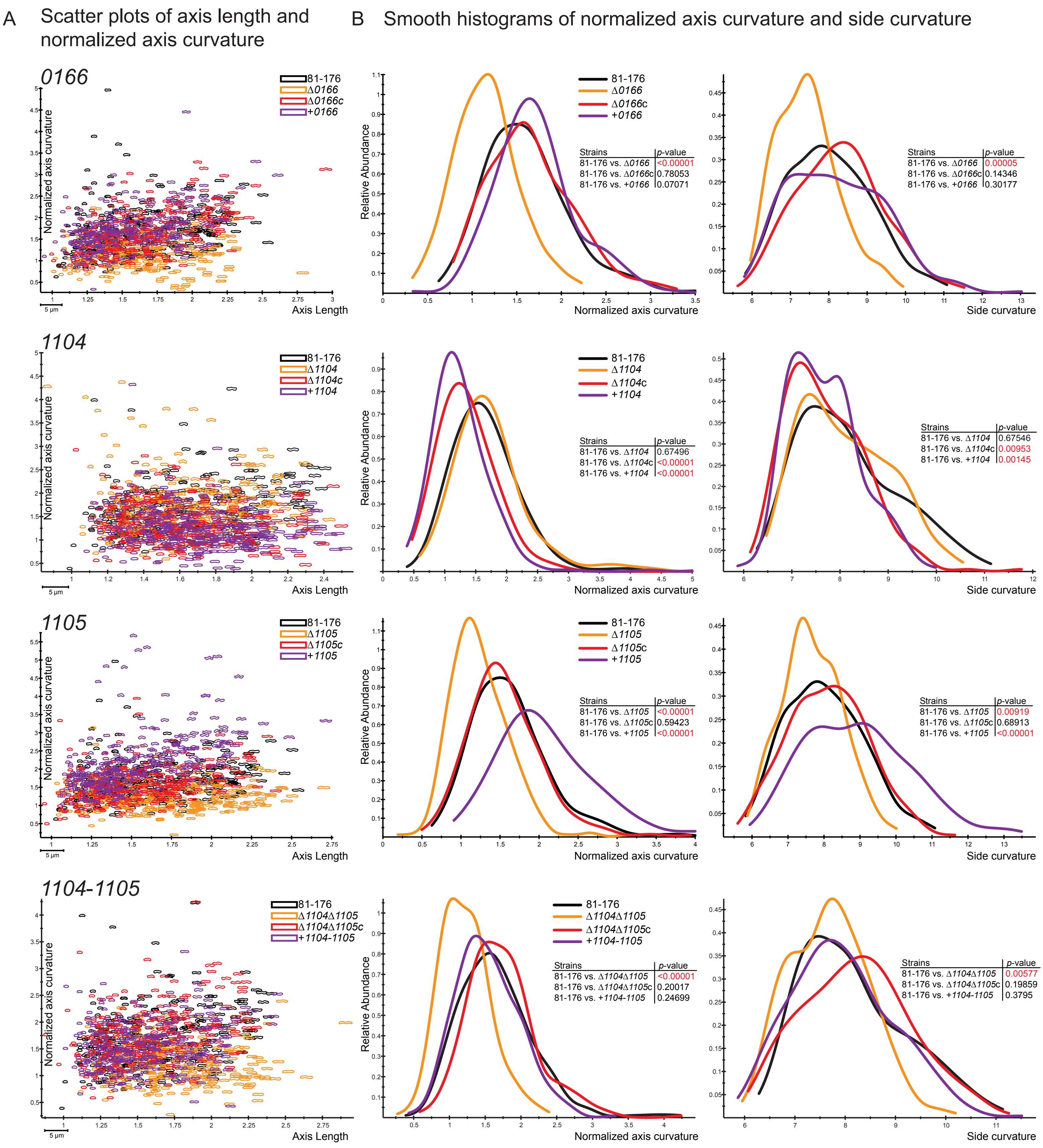
A Scatter plots of axis length and normalized axis curvature
B Smooth histograms of normalized axis curvature and side curvature
0166
81-176
Δ0166
Δ0166c
+0166
Strains | p-value
81-176 vs. Δ0166 | <0.00001
81-176 vs. Δ0166c | 0.78053
81-176 vs. +0166 | 0.07071
Strains | p-value
81-176 vs. Δ0166 | 0.00005
81-176 vs. Δ0166c | 0.14346
81-176 vs. +0166 | 0.30177
1104
81-176
Δ1104
Δ1104c
+1104
Strains | p-value
81-176 vs. Δ1104 | 0.67496
81-176 vs. Δ1104c | <0.00001
81-176 vs. +1104 | <0.00001
Strains | p-value
81-176 vs. Δ1104 | 0.67546
81-176 vs. Δ1104c | 0.00953
81-176 vs. +1104 | 0.00145
1105
81-176
Δ1105
Δ1105c
+1105
Strains | p-value
81-176 vs. Δ1105 | <0.00001
81-176 vs. Δ1105c | 0.59423
81-176 vs. +1105 | <0.00001
Strains | p-value
81-176 vs. Δ1105 | 0.00919
81-176 vs. Δ1105c | 0.68913
81-176 vs. +1105 | <0.00001
1104-1105
81-176
Δ1104Δ1105
Δ1104Δ1105c
+1104-1105
Strains | p-value
81-176 vs. Δ1104Δ1105 | <0.00001
81-176 vs. Δ1104Δ1105c | 0.20017
81-176 vs. +1104-1105 | 0.24699
Strains | p-value
81-176 vs. Δ1104Δ1105 | 0.00577
81-176 vs. Δ1104Δ1105c | 0.19859
81-176 vs. +1104-1105 | 0.3795
Normalized axis curvature
Axis Length
5 μm
Relative Abundance
Side curvature

Figure S5

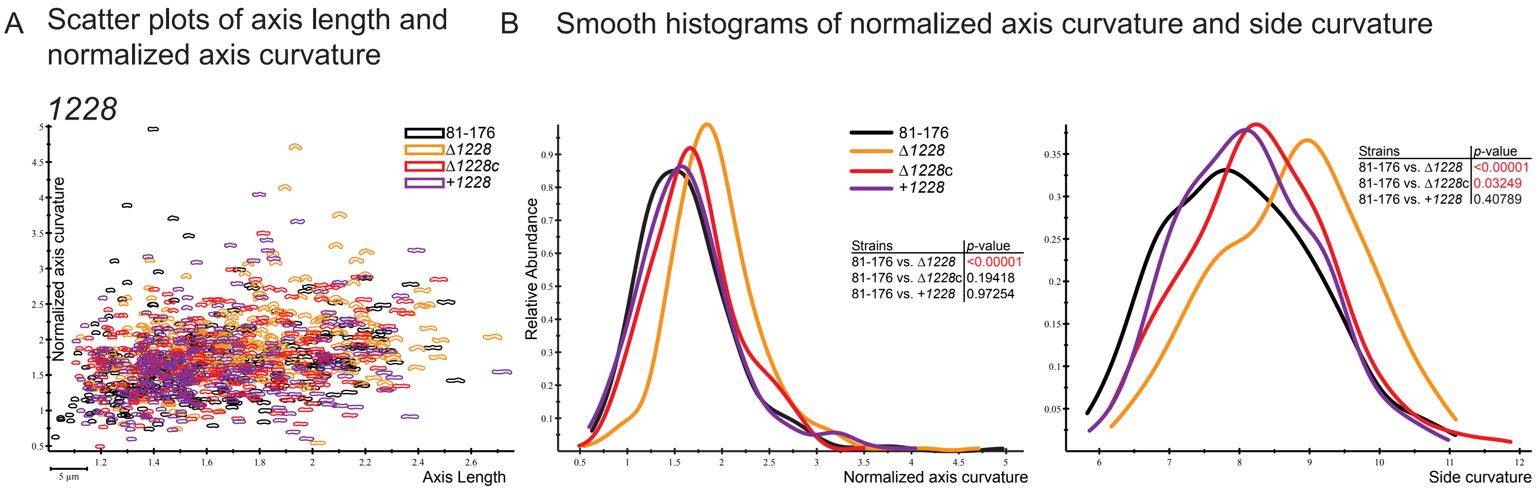

Figure S5. CellTool analysis to examine differences in cell curvature in *C. jejuni 0166, 1104, 1105*, and *1228* mutant, complement and overexpressor strains. The curvature of cell contours of mutant (Δ*0166*, Δ*1104*, Δ*1105*, Δ*1104*Δ*1105,* and Δ*1228*), complemented (Δ*0166*c, Δ*1104*c, Δ*1105*c, Δ*1104*Δ*1105*c, and Δ*1228*c) and overexpressing (+*0166*, +*1104*, +*1105*, +*1104-1105* and +*1228*) strains was determined by CellTool analysis from binary images extracted from DIC images used in Fig. 3. **A**, scatterplot of cell contours with axis length plotted on the x-axis and normalized axis curvature on the y-axis. **B**, smooth histogram of normalized axis curvature and side curvature relative to abundance. Normalized axis curvature measures the normalized curvature of the central axis and side curvature the normalized curvature along the contour itself excluding the poles. The analyses are grouped by gene: *0166*, *1104*, *1105*, *1104-1105*, and *1228*. The *p*-values comparing each strain to wild type were determined by Kolmogorov-Smirnov statistics and are shown as an inset to each graph. Values in red highlight statistically significant comparisons with p-values <0.05.

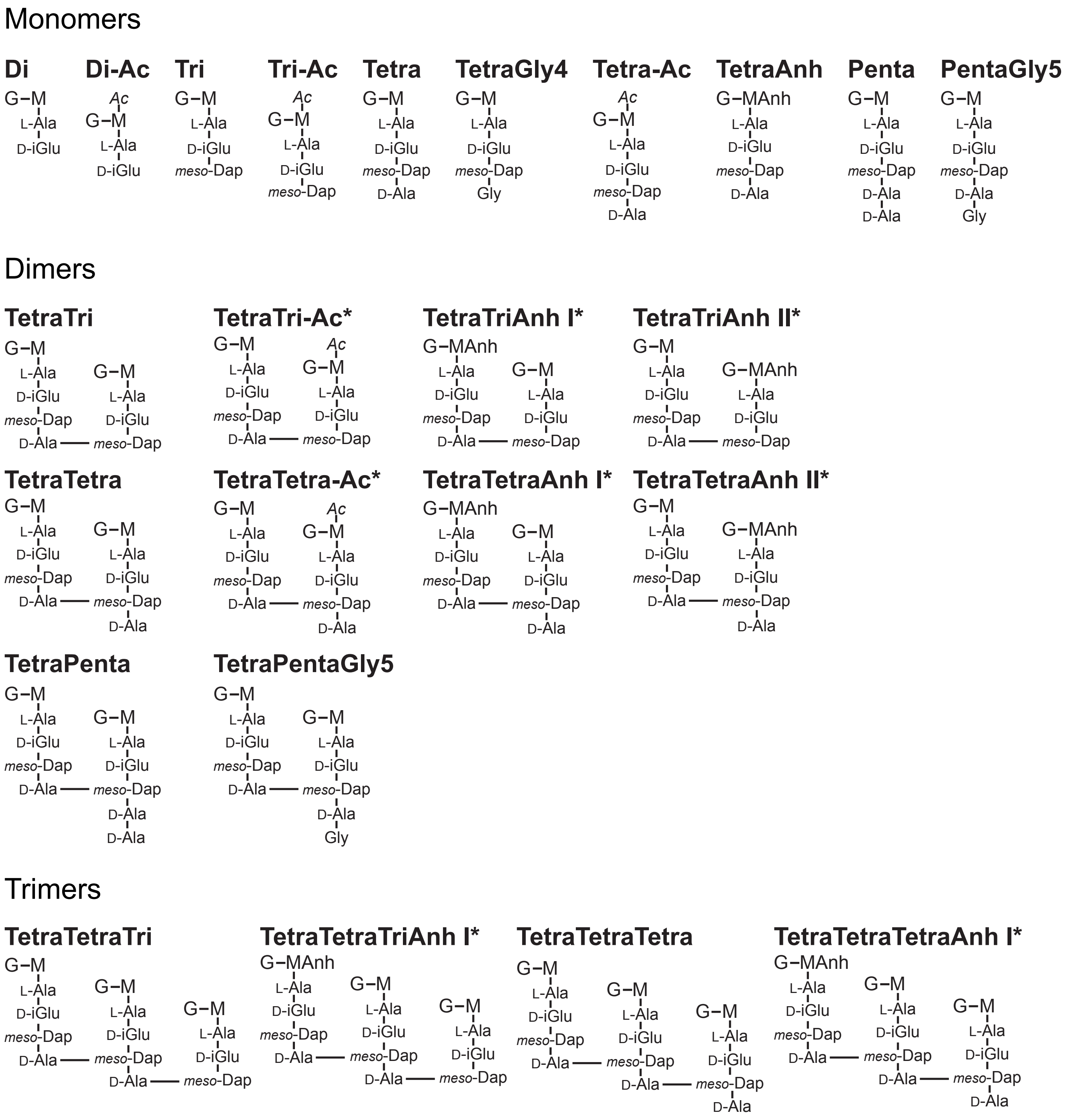

Figure S6. Muropeptide structures. Purified PG was digested with cellosyl, and the resulting muropeptides were reduced with sodium borohydride and separated on a Prontosil 120-3-C18 AQ reverse-phase column. Shown are the structures of the main muropeptide peak fractions analyzed by LTQ-FT-MS. G, N-acetylglucosamine; M, reduced N-acetylmuramic acid; L-Ala, L-alanine; D-iGlu, D-isoglutamic acid; D-Glu, D-glutamic acid; meso-DAP, meso-diaminopimelic acid; Gly. Glycine; Ac, O-acetyl groups at the C-6 hydroxyl group of MurNAc; Anh, 1,6-anhydro group at MurNAc. The asterisk (*) indicates that it is not known on which MurNAc residue the modification occurs.

References

An, D.R., Kim, H.S., Kim, J., Im, H.N., Yoon, H.J., Yoon, J.Y., et al. (2015). Structure of Csd3 from *Helicobacter pylori*, a cell shape-determining metallopeptidase. *Acta Crystallogr D Biol Crystallogr* 71(Pt 3)**,** 675-686. doi: 10.1107/S1399004715000152.

Cameron, A., and Gaynor, E.C. (2014). Hygromycin B and apramycin antibiotic resistance cassettes for use in *Campylobacter jejuni*. *PLoS One* 9(4)**,** e95084. doi: 10.1371/journal.pone.0095084.

Deng, X., Gonzalez Llamazares, A., Wagstaff, J.M., Hale, V.L., Cannone, G., McLaughlin, S.H., et al. (2019). The structure of bactofilin filaments reveals their mode of membrane binding and lack of polarity. *Nat Microbiol* 4(12)**,** 2357-2368. doi: 10.1038/s41564-019-0544-0.

Frirdich, E., Biboy, J., Adams, C., Lee, J., Ellermeier, J., Gielda, L.D., et al. (2012). Peptidoglycan-modifying enzyme Pgp1 is required for helical cell shape and pathogenicity traits in *Campylobacter jejuni*. *PLoS Pathog* 8(3)**,** e1002602. doi: 10.1371/journal.ppat.1002602.

Frirdich, E., Biboy, J., Huynh, S., Parker, C.T., Vollmer, W., and Gaynor, E.C. (2017). Morphology heterogeneity within a *Campylobacter jejuni* helical population: the use of calcofluor white to generate rod-shaped C. jejuni 81-176 clones and the genetic determinants responsible for differences in morphology within 11168 strains. *Mol Microbiol* 104(6)**,** 948-971. doi: 10.1111/mmi.13672.

Karlyshev, A.V., and Wren, B.W. (2005). Development and application of an insertional system for gene delivery and expression in *Campylobacter jejuni*. *Appl Environ Microbiol* 71(7)**,** 4004-4013.

Korlath, J.A., Osterholm, M.T., Judy, L.A., Forfang, J.C., and Robinson, R.A. (1985). A point-source outbreak of campylobacteriosis associated with consumption of raw milk. *J Infect Dis* 152(3)**,** 592-596.

Menard, R., Sansonetti, P.J., and Parsot, C. (1993). Nonpolar mutagenesis of the *ipa* genes defines IpaB, IpaC, and IpaD as effectors of *Shigella flexneri* entry into epithelial cells. *J Bacteriol* 175(18)**,** 5899-5906.

Shi, C., Fricke, P., Lin, L., Chevelkov, V., Wegstroth, M., Giller, K., et al. (2015). Atomic-resolution structure of cytoskeletal bactofilin by solid-state NMR. *Sci Adv* 1(11)**,** e1501087. doi: 10.1126/sciadv.1501087.

Stahl, M., Frirdich, E., Vermeulen, J., Badayeva, Y., Li, X., Vallance, B.A., et al. (2016). The Helical Shape of *Campylobacter jejuni* Promotes *In Vivo* Pathogenesis by Aiding Transit through Intestinal Mucus and Colonization of Crypts. *Infect Immun* 84(12)**,** 3399-3407. doi: 10.1128/IAI.00751-16.

Vasa, S., Lin, L., Shi, C., Habenstein, B., Riedel, D., Kuhn, J., et al. (2015). beta-Helical architecture of cytoskeletal bactofilin filaments revealed by solid-state NMR. *Proc Natl Acad Sci U S A* 112(2)**,** E127-136. doi: 10.1073/pnas.1418450112.

Yao, R., Alm, R.A., Trust, T.J., and Guerry, P. (1993). Construction of new *Campylobacter* cloning vectors and a new mutational cat cassette. *Gene* 130(1)**,** 127-130.
